# Nucleotide composition affects codon usage toward the 3'-end

**DOI:** 10.1371/journal.pone.0225633

**Published:** 2019-12-04

**Authors:** Fouad Zahdeh, Liran Carmel

**Affiliations:** 1 Department of Genetics, The Alexander Silberman Institute of Life Sciences, Faculty of Science, The Hebrew University of Jerusalem, Edmond J. Safra Campus, Givat Ram, Jerusalem, Israel; 2 Hereditary Research Lab, Life Sciences Department, Bethlehem University, Bethlehem, Palestinian authority; Tel Aviv University, ISRAEL

## Abstract

The 3’-end of the coding sequence in several species is known to show specific codon usage bias. Several factors have been suggested to underlie this phenomenon, including selection against translation efficiency, selection for translation accuracy, and selection against RNA folding. All are supported by some evidence, but there is no general agreement as to which factors are the main determinants. Nor is it known how universal this phenomenon is, and whether the same factors explain it in different species. To answer these questions, we developed a measure that quantifies the codon usage bias at the gene end, and used it to compute this bias for 91 species that span the three domains of life. In addition, we characterized the codons in each species by features that allow discrimination between the different factors. Combining all these data, we were able to show that there is a universal trend to favor AT-rich codons toward the gene end. Moreover, we suggest that this trend is explained by avoidance from forming RNA secondary structures around the stop codon, which may interfere with normal translation termination.

## Introduction

Codon usage bias (CUB) is the name coined for the well-known observation that synonymous codons are used at different frequencies in a genome. The codons that are more frequently used are denoted *optimal codons*. Different organisms may show different CUB, in the sense that they have different optimal codons. It has been observed that optimal codons tend to be over-represented in highly expressed genes [1, 2] and to correspond to high copy number of cognate tRNAs [3–6]. Over the years, there have been many attempts to identify the factors that are at the base of CUB. Of them, two leading explanations suggest selective constraints. The first is termed *translation efficiency* and it asserts that codons with higher density of cognate tRNAs will be translated faster, thus a coding sequence (CDS) made predominantly of optimal codons would be translated more efficiently [1, 7, 8]. The second is termed *translation accuracy* [9–11] and it asserts that less frequent codons are more prone to translational errors due to increased competition from more abundant, near-cognate tRNAs [3, 12, 13]. Both types of selection may be in action in each particular species [14, 15], but their relative contribution is typically unknown [9]. In some eukaryotes, such as human and drosophila, it had been claimed that the effect of translation efficiency is weak [2, 6, 7, 16, 17]. Similar claim has been made also in some prokaryotes such as *H*. *pylori*, where translation efficiency seems to have no significant contribution to CUB [18]. In addition to these presumed selective forces, many other factors have been suggested to effect CUB, including biased gene conversion [19], effective population size and evolutionary history [10, 20–22], genome size [7], mutation-selection balance [6, 10, 16], Hill-Robertson effect [23–27], and mRNA secondary structure [28–34].

In addition to the genome-wide CUB, it has been noticed that codon usage shows spatial patterns along the CDS. While CUB at the CDS start (5'-end) has been extensively studied [25, 29, 31–35], less is known about its behavior towards the CDS end. Tuller *et al*. [35] reported that codons at the 3'-end are translationally inefficient (hereinafter, simply inefficient) in many species, especially in eukaryotes. This finding supports previous work by Eyre-walker who found a decrease in the frequency of efficient codons along the last 20 codons of *E*. *coli* genes [36]. However, this observation does not seem to universally hold. For example, Qin *et al*. measured 3’-end CUB in four prokaryotes and two eukaryotes, and could not find a consistent trend [25].

Translation accuracy, and more specifically selection against nonsense errors, is thought to be a key factor affecting the spatial distribution of CUB [25, 37]. It was suggested that the cost of nonsense errors increases when translation progresses, until it peaks near the 3’-end [29, 38, 39]. Accordingly, it is expected that selection to minimize nonsense errors is stronger near the stop codon [8, 25, 37]. Qin *et al*. [25] tested this by studying spatial CUB along the entire CDS, and concluded that selection against nonsense errors dominates in prokaryotes and yeast. Another version of selection against nonsense errors in eukaryotes was reported by Cusack *et al*. [40]. They claimed that the selection regime along the CDS in eukaryotes is different than in prokaryotes, as the eukaryotic mRNA surveillance mechanism known as nonsense-mediated mRNA decay (NMD) targets for degradation transcripts that harbor premature stop codons. As the main trigger to NMD is the presence of an exon-exon junction downstream to the stop codon [41], all nonsense errors except those in the last exon, do not yield functional transcripts and are therefore not highly deleterious. Cusack *et al*. studied a group of codons that are one substitution away from stop codons, called fragile codons, which should show this unique selection regime more strongly. As expected, fragile codons were shown to be depleted in the last exon of multi-exon genes in human, but not in other exons. It is not discussed in Cusack *et al*.’s paper, but even along the last exon the selection regime on fragile codons is not expected to be even. The reason is that the deleterious effect of nonsense errors is expected to decrease towards the 3’-end, as slightly truncated proteins usually retain most of their functionality.

Another factor that was suggested to be important in shaping spatial CUB is selection against RNA folding. Rocha *et al*. found that there is a tendency toward GC depletion at both the 5’ and the 3’ gene termini in *B*. *subtilis* [31]. They suggested that the bias at the 3’-end decreases the propensity to form stable RNA secondary structures, and hence lowers the probability of interference with normal translation termination and the recruitment of release factors. A similar observation was made in an earlier study by Erye-Walker [36], who reported an increase in A-ending codons and a decrease in G-ending codons toward the 3’-end of *E*. *coli* genes. Because many genes in *E*. *coli* overlap the CDS or the Shine-Dalgarno sequence of another gene in the opposite strand, Erye-Walker explained this bias by the need to avoid RNA secondary structures near the transcription start site of the opposing gene. However, further studies showed that the bias likely relates to the gene end, rather than to the start of the opposing gene. Katz *et al*. [42] examined the potential to form secondary structures at the 5’ and the 3’ termini of genes in *E*.*coli* and yeast. In *E*. *coli*, the propensity to form RNA secondary structures was evenly distributed across the CDS, and specifically was about equal at both gene termini. In yeast, however, the propensity to form RNA secondary structures was found to be lower at the 3’-end than at the 5’-end. However, the universality of this factor was questioned as well. Qin *et al*. studied the effect of various factors on CUB near the gene end in yeast and fruit fly, and showed that the spatial variation in CUB is inconsistent with the GC-content variation across the gene [25].

As of today, it is still unclear which of the above models, or a combination thereof, best explains the 3’-end CUB patterns. All models are partly supported, and it may be that different models better describe the CUB in different species. Another difficulty is that the different models have very similar predictions, because of strong dependencies between the relevant codon features. For example, AT-rich codons also tend to be inefficient [34] and fragile [40]. Such dependencies have been accounted for in some works on CUB. For instance, based on the fact that the transcription initiation site in many species experiences a selection against mRNA folding [32, 33], and because inefficient codons were found to be AT-rich, Bentele *et al*. proposed that the reduction in translation efficiency at the gene 5’-end is a side effect of the selection against mRNA folding [34]. However, to the best of our knowledge, no work has compared the different models relevant to the CDS end in a way that accounts for these dependencies. This is the task we wish to accomplish in this work.

Another difficulty in direct comparison of the different models stems from the lack of uniformity in measuring CUB levels. The *codon adaptation index* (CAI) is one of the most popular measures of CUB, that attempts to directly account for the observation that highly expressed genes tend to use more efficient codons [43]. A similar measure is called *tRNA adaptation index* (tAI), and it attempts to normalize codon usage to the background tRNA pool [44]. Other measures, such as the *effective number of codons* (ENC) [45] or the *relative synonymous codon usage* (RSCU) [46], estimate the deviation of codon usage from their expected usage under the null uniform distribution.

A major obstacle in studying spatial CUB is that none of the existing measures is position-dependent, thus all previous works measured an overall, rather than spatial, CUB, even when focusing on a particular region of the CDS. To circumvent this, Qin *et al*. aligned subsets of genes to create super-sequences, and then computed the *ENC* index for each position [25]. Tuller *et al*. used a similar approach, which they termed *local tAI* [35]. In local tAI, genes were aligned either from their 3'-end or from their 5'-end, and the average tAI index was computed at each position. Hockenberry *et al*. [47] studied codon usage bias in *E*. *coli* using a position-dependent model. They partitioned the sequence into bins of equal number of codons and defined the positional dependency (pD) of each bin as the χ^2^ statistics based on the observed frequency of individual codons at the specific bin and their expected frequency derived from a random synonymous shuffling algorithm. Such approaches assume that all positions have the same relative weight in the computation of the index, so it is technically a local measure of the otherwise global index at different gene regions, and not a full fledge position-dependent index.

Here, we develop a position-dependent codon usage index that we dub *relative spatial codon abundance* (RSCA). We used RSCA to characterize the spatial CUB near the 3’-end of 91 species, representing all three domains of life. For each species we tested how much of the 3’-end CUB is explained by each of the three main models: selection against translation efficiency, selection for translation accuracy, and selection against mRNA folding. In all species, we found a strong support to the notion that 3’-end CUB stems from avoidance of RNA secondary structures, suggesting that this is a universal selective force.

## Results

### Measuring spatial codon usage bias towards the gene end

We developed a measure, called relative spatial codon abundance (RSCA) that measures how over- or under-represented a codon is at each position along the gene. The RSCA is represented by a matrix $R_{\alpha }^{i}$, which computes the relative spatial abundance of codon *i* at position *α*, measured as the codon number from the gene end (so that *α* = 1 corresponds to the last codon before the stop codon, *α* = 2 corresponds to the codon just before it, and so on.) $R_{\alpha }^{i}=1$ means that the frequency of codon *i* at position *α* is as expected from the overall relative abundance of this codon in the genome. $R_{\alpha }^{i}>1$ means that the codon is over-represented at this position, whereas $R_{\alpha }^{i}<1$ means that the codon is under-represented at that position (see Methods for a complete definition).

We computed RSCA for 91 species representing all three domains of life (S1 Table). Importantly, the list includes three species, *M*. *pneumonia*, *M*. *florum and U*. *parvum*, that use a modified genetic code, allowing to test various connections between the structure of the genetic code and the abundance of codons near the gene end. We noticed that the RSCA in the last position before the stop codon (*α* = 1) usually deviates from its values in the preceding positions. This likely reflects special and strong constraints on the penultimate codon to increase termination efficiency [48]. Consequently, we ignored this position from further analyses, and instead focused on more gradual changes to RSCA values, occurring over at least a few codons.

### Multiple strategies for grouping codons

To evaluate the contribution of each of the three models on the RSCA at the CDS 3’-end, we characterized each codon by a series of informative features. Features informative for selection against mRNA folding are the codon GC content and the content of its third nucleotide (wobble position). A feature informative for selection against translation efficiency is the cognate tRNA copy number. Finally, a feature informative for selection for translation accuracy is the codon fragility. Therefore, we used four different classification schemes, dividing all sense codons (excluding those that have no synonymous codons) into mutually exclusive groups based on the value of the relevant features:

1. Wobble position. We divided the sense codons into four groups, based on the identity of their third nucleotide: *A-ending* codons, *T-ending* codons, *C-ending* codons, and *G-ending* codons.
2. GC content. We grouped the codons based on their total GC content, from 0 (the codon harbors neither C nor G) to 3 (the codon harbors only Cs and Gs).
3. Fragility. As explained in the Introduction, fragile codons are codons that are one substitution away from a stop codon. We have further divided fragile codons into *replaceable* codons, which code for an amino acid that is coded by at least one non-fragile codon, and *non-replaceable* codons, which code for an amino acid that is exclusively coded by fragile codons. The reason for this distinction is that if there is a selection against fragile codons, it is much easier to avoid the usage of replaceable codons, but far harder for non-replaceable ones. Overall, this classification scheme divides all sense codons into three groups: non-fragile, replaceable, and non-replaceable.
4. Efficiency. Codons were binned to three groups based on their relative adaptiveness value, which is an estimate of the abundance of the corresponding tRNAs [7]. Higher value of the relative adaptiveness indicates higher abundance of tRNAs that recognize the codon, and suggests that the codon is more translationally efficient. The three groups are denoted *inefficient* codons, *moderately efficient* codons, and *efficient* codons.

A major obstacle in judging which feature is most explanatory of 3’-end spatial CUB patterns is that the different classification schemes are not independent: Efficiency significantly depends on fragility in 18 species, on wobble position in 51 species, and on GC content in eight species. (*P*≤0.05, FDR-corrected *χ*^2^ independence test; S2 Table); fragility significantly depends on wobble position in the standard genetic code (*P* = 0.03) but not in the Mycoplasma genetic code (*P* = 0.11). These results show that different codon features may be strongly correlated, and that these correlations may be species-specific. These connections must be taken into account, and may lay behind part of the disagreements between previous studies.

### Measuring RSCA towards the gene end for codon groups

To compute the spatial patterns displayed collectively by a group of codons *S*, we developed a group RSCA measure, denoted $R_{\alpha }^{S}$ (see Methods). Like before, $R_{\alpha }^{S}=1$ means that the frequency of the codons in *S* at position *α* is as expected from their overall relative abundance, $R_{\alpha }^{S}>1$ means that they are over-represented at this position, and $R_{\alpha }^{S}<1$ means that they are under-represented at that position (see Methods).

We tested this measure by applying it to permuted datasets, in which we kept the overall codon usage bias, but erased positional variance. To this end, we picked four genomes (*A*. *pernix*, *E*. *coli*, *H*. *sapiens*, and *M*. *pneumonia*) with a representative from each of the three domains of life, as well as a representative (*M*. *pneumonia*) for non-standard genetic code. In each permutation, we replaced each codon by a synonymous codon according to its genomic frequency. For example, Alanine is coded by ‘GCA’, ‘GCC’, ‘GCG’, and ‘GCT’ codons. Their frequencies in *E*. *coli* are 0.22, 0.27, 0.34, and 0.17, respectively. We scanned the *E*. *coli* genome and replaced each Alanine codon by a synonymous codon (including the same codon) with a probability that equals the synonymous codon genomic frequency. At the end of this process, each of these codons appears in roughly the same frequency as in the original genome, but positional bias is erased. We performed this process 100 times for each of the four genomes, and obtained, as expected, group RSCA scores close to 1 along the last 50 codons of the gene in all tested genomes (S1–S4 Figs; S3 Table).

**Fig 1:**
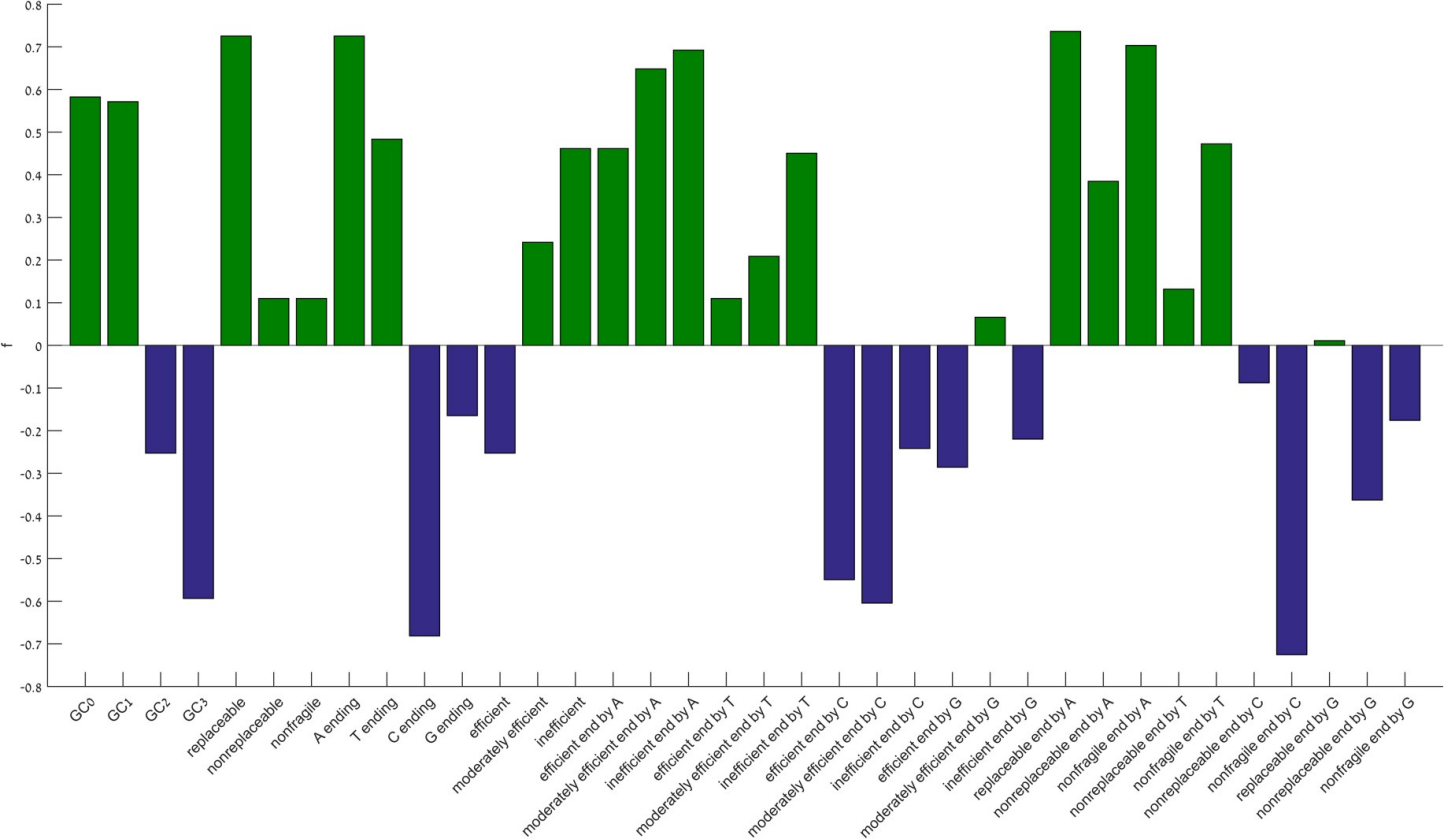
Preference of codon features towards the gene end. For each codon group we calculated the sum of scores (S5 Table) divided by the total number of species, *f*. Green bars mark features in which group RSCA significantly increase, blue bars mark features that are significantly decreased toward 3’-end.

We computed the group RSCA score for each group of codons in each of the classification schemes, and for each of the species, along the last 50 codons of genes. To measure how significantly $R_{\alpha }^{S}$ increases or decreases toward the 3’-end, we fit it to a linear model. As the exact position where RSCA starts to deviate from one changes across species and domains (details below), the linear model was evaluated separately at the last 10, 20, and 30 codons (three regions). For each model we computed the adjusted *R*^2^, slope *β*, and FDR-adjusted p-value *P*. *P*<0.05 and *β*>0 at any region implies significant increase in the representation of the codon towards the 3’-end. *P*<0.05 and *β*<0 at any region implies significant decrease in the representation of the codon towards the 3’-end (S4 Table). To summaries the linear regression model across multiple species, we scored a codon group in a species by 1 if it shows significant increase, by -1 if it shows significant decrease, and by 0 if the linear model is insignificant in all three regions (S5 Table). This scoring system cancels out the contribution of species which have opposing trends for the same codon group. Finally, each codon group was assigned with a value *f*, which is the sum of scores across all species, divided by the number of species (Fig 1). Codons that are AT-rich, A-ending, and replaceable are the top over-represented groups, while C-ending and GC-rich are the top under-represented codon groups. For further analysis, see below.

### A-rich codons are preferred towards the gene end

**Fig 2:**
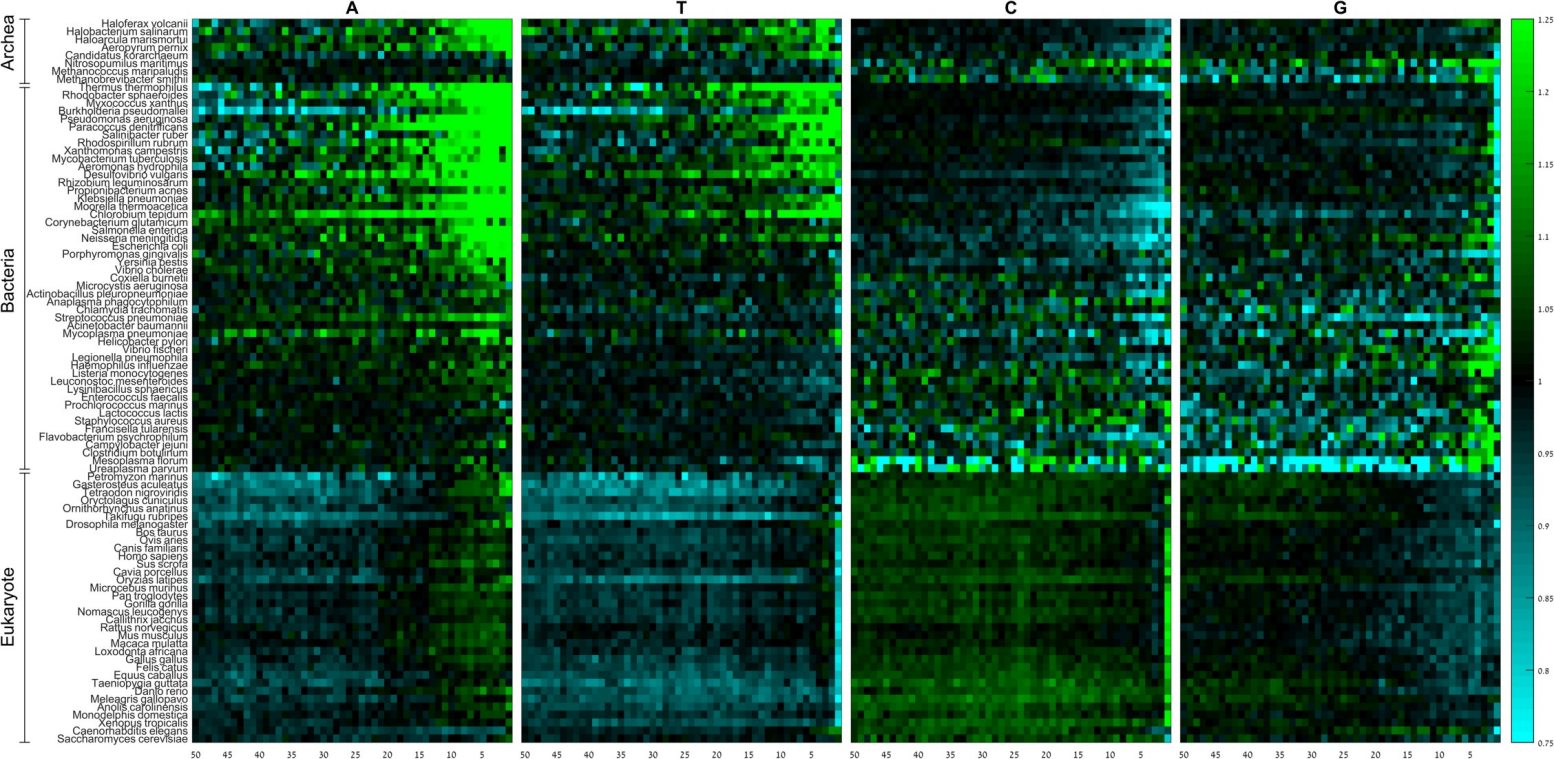
Group RSCA scores of A-ending, T-ending, C-ending, and G-ending codons along the last 50 codons of the gene. Rows denote species, columns denote positions. Species within domains are sorted from high (top) to low (bottom) GC-content.

When classifying codons by the content of their wobble position, we observed that A-ending codons are preferentially used towards the gene end in a striking majority of species (Fig 2; S4 and S5 Tables). The few exceptions include the eukaryote *C*. *elegans*, and the archaeon *N*. *martimus*, both showing significant under-representation of A-ending codons. The yeast *S*. *cerevisiae* and a few prokaryotes show neither under-representation nor over-representation of A-ending codons near the gene end (see Discussion). The extent of this spatial pattern is shared across domains, with A-ending codons showing preferential usage along the last 10–15 codons. Interestingly, only in eukaryotes A-ending codons tend to be highly under-represented also along the preceding region (positions 15/20-50). T-ending codons do not show a consistent trend, although they are markedly under-represented in eukaryotes along most of the last 50 codons of the gene, with the exception of positions 3–5. The spatial patterns of C-ending codons are almost the exact opposite of those for T-ending codons, but the under-representation in prokaryotes is much more pronounced than in eukaryotes. The spatial patterns of G-ending codons show significant contrast between domains. While they seem to be over-represented in positions 3–6 in some prokaryotes, they tend to be under-represented along the last 15–30 codons in eukaryotes.

In summary, spatial patterns of codons ending with a particular nucleotide show different trends between domains, except for A-ending codons that are over-represented in the vast majority of studied species from all the domains.

**Fig 3:**
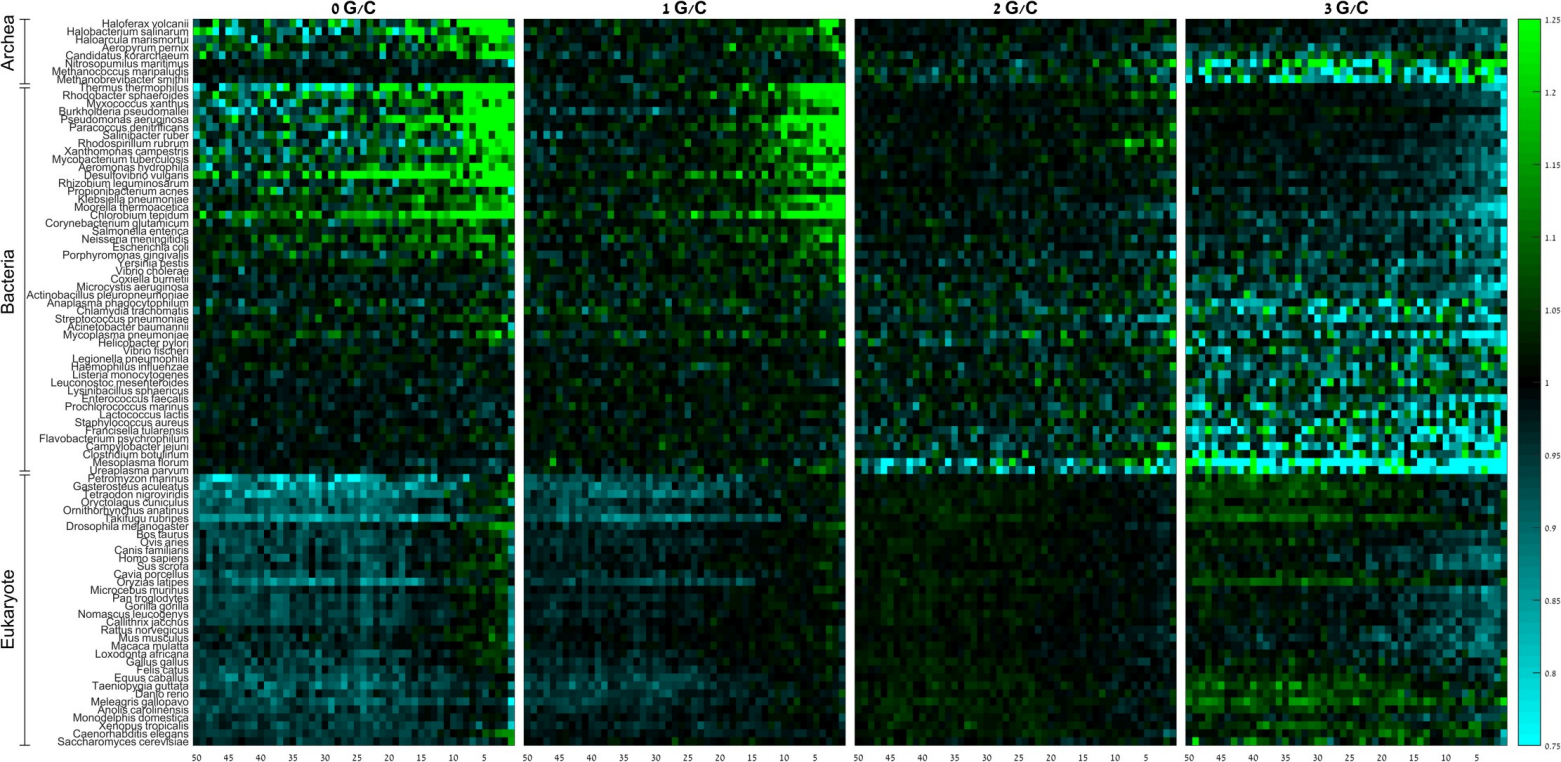
Group RSCA scores of codons containing 0, 1, 2, and 3 G/C nucleotides along the last 50 codons of the gene. Rows denote species, columns denote positions. Species within domains are sorted from high (top) to low (bottom) GC-content.

Similar results are obtained if we group codons based on their GC content (Fig 3; S4 and S5 Tables). Almost all examined species show strong preference to A/T-rich codons at their gene termini, along the last 5–10 codons of the gene. These observations are in agreement with previous works [31, 36, 42], that attributed this trend to a pressure to avoid mRNA folding at the vicinity of the stop codon in order to guarantee efficient translation termination.

We repeated the analyses on triplets that were formed by introducing +1nt and +2nt frameshifts to the coding region. By introducing a +1nt frameshift, namely looking at the content of the first codon position in the original reading frame, we found that RSCA patterns of A, T, C, and G-ending triplets are generally similar to the patterns we observed for the original reading frame: A-ending triplets are preferred while G-ending triplets are avoided toward the 3’-end (S5 Fig). Interestingly, species that deviate from the general preference to A-ending codons, such as *C*. *elegans* and *N*. *maritimus* (see Figs 2 and 3), have similar patterns to the rest of species in the +1nt triplets, especially at the last 5–10 codons. However, after introducing +2nt frameshift (i.e., looking at the content of the second codon position in the original reading frame) triplets grouped by wobble position show completely different patterns (S6 Fig). C-ending triplets are under-represented in prokaryotes, especially at the last 15 triplets, and A-ending triplets are slightly over-represented along the last 10–20 codons in many species. G-ending and T-ending triplets show mixed patterns.

A similar pattern is observed when grouping codons by GC richness. For +1nt frameshift, the patterns are similar to the original reading frame, where GC-rich triplets (3/3 codons) are avoided and AT-rich triplets (0/3 codons) are preferred toward the 3’-end (S7 Fig). This pattern is similar, but less pronounced for +2nt frameshift (S8 Fig).

Taken together, these results suggest that there is a strong selection against G/C and a preference to A at the first and third codon positions, but not at the second codon position. Given that any mutation in the second codon position is nonsynonymous, these findings are compatible with the notion that these trends in CUB towards the gene end are independent of selection forces at the amino acid level. This also suggests that the trends we observe generally characterize nucleotide composition in codons, and are not specific to particular position along the codon.

### Efficiency is not a major factor determining codon usage bias near the gene end

**Fig 4:**
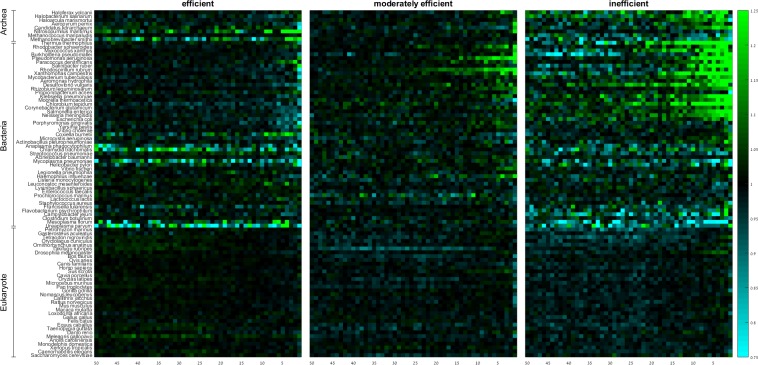
Group RSCA scores of efficient, moderately efficient, and inefficient codons along the last 50 codons of the gene. Rows denote species, columns denote positions. Species within domains are sorted from high (top) to low (bottom) GC-content.

Next, we wanted to check the effect of codon efficiency on the spatial codon usage bias near the stop codon by computing the group RSCA score of codons based on their adaptiveness values (Fig 4). In the majority of the examined prokaryotes, and in particular in those with high to moderate GC-content, inefficient and moderately efficient codons are preferentially used towards the gene end, consistent with previous reports [35, 36]. These codons tend to be under-represented along the entire region in eukaryotes except for the last 5 codons (Fig 4). Efficient codons generally show the opposite pattern.

**Fig 5:**
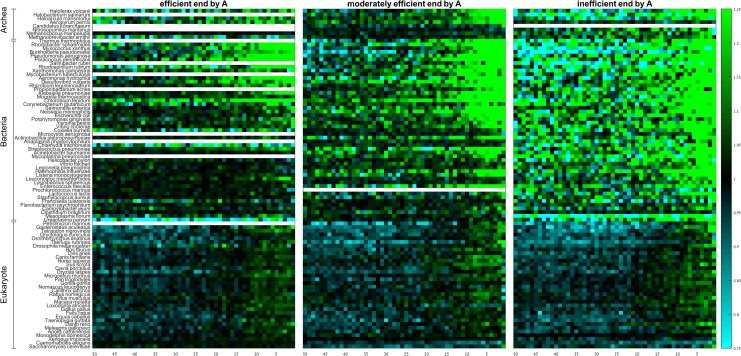
Group RSCA scores of efficient, moderately efficient, and inefficient codons that end by A along the last 50 codons of the gene. Rows denote species, columns denote positions. Species where none of the codons in a particular efficiency group end by A (missing data) are shown as white stripes. Species within domains are sorted from high (top) to low (bottom) GC-content.

As codon efficiency significantly depends on wobble position in more than half of the species (S2 Table), and because inefficient codons are known to be AT rich [34], we wished to test whether the spatial patterns of inefficient codons prevail after we control for the content of their wobble position. To this end, we subdivided the inefficient, moderately efficient, and efficient codon groups into codons that end by A (Fig 5; S4 and S5 Tables), T, C, and G (S9–S11 Figs; S4 and S5 Tables). This analysis shows that the spatial patterns towards the gene ends are predominantly dictated by the wobble position rather than by codon efficiency. The patterns of codons with the same efficiency depend on whether they end by A, T, G, or C, and codons ending with the same nucleotide show similar patterns regardless of their efficiency. To further confirm this we used the (FDR-corrected) Kruskal-Wallis test in the last 15 positions to check whether the distribution of the RSCA values of codons ending by A/T and of codons ending by C/G are affected by the codon efficiency (S6 and S7 Tables). The results show that RSCA values do not depend on the efficiency of the codon, as long as we control for the content of its wobble position. We conclude that selection against translation efficiency plays no significant role in shaping the codon usage bias at the gene 3’-end.

### Fragility is not a major factor determining codon usage bias near the gene end

**Fig 6:**
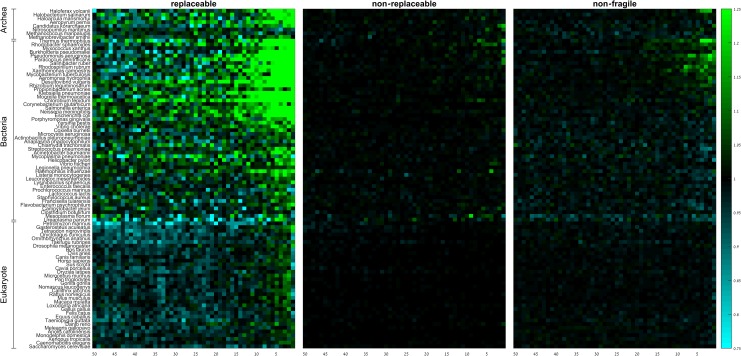
Group RSCA scores of replaceable, non-replaceable, and non-fragile codons along the last 50 codons of the gene. Rows denote species, columns denote positions. Species within domains are sorted from high (top) to low (bottom) GC-content.

We computed group RSCA scores for codon fragility (Fig 6; S4 and S5 Tables). The results show a strong preference to replaceable codons along the last 5–10 codons, especially in prokaryotes. Non-replaceable codons show negligible spatial preferences. Non-fragile codons exhibit a weak tendency to be over-represented along the last 10–20 codons in high GC-content prokaryotes but a weak tendency to be under-represented along the last 10–20 codons in prokaryotes with low to moderate GC-content.

**Fig 7:**
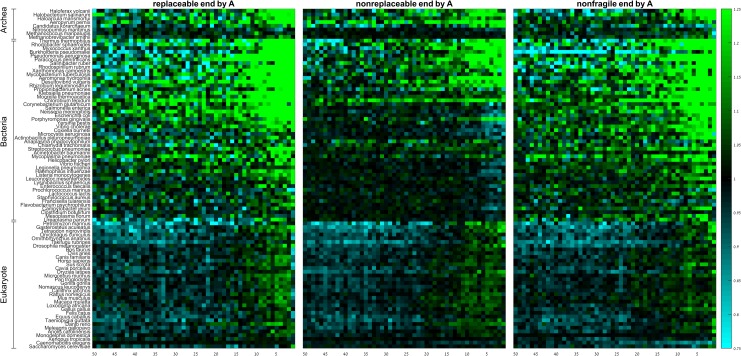
Group RSCA scores of replaceable, non-replaceable, and non-fragile codons that end by A along the last 50 codons of the gene. Rows denote species, columns denote positions. Species within domains are sorted from high (top) to low (bottom) GC-content.

Replaceable codons are enriched for codons that end by A (71% of the replaceable codons, but only 27% of the non-replaceable and 14% of the non-fragile). Moreover, fragility strongly depends on wobble position in species that utilize the standard genetic code (*P* = 0.03; *χ*^2^ independence test). Therefore, we wanted to test whether their spatial patterns are a simple reflection of the content of their wobble position. Similar to what we did for codon efficiency, we subdivided the replaceable, non-replaceable and non-fragile codons according to the content of their wobble position into A- (Fig 7; S4 and S5 Tables), T-, C-, and G-ending codons (S12–S14 Figs; S4 and S5 Tables), and tested whether fragility affects the distribution of the RSCA values (S8 and S9 Tables). The results demonstrate that replaceable, non-replaceable, and non-fragile codons ending by A behave similarly irrespective to their fragility, and that the distribution of RSCA values is independent on the fragility of the codon. Exceptions include some prokaryotes, especially AT-rich ones, that show weak under-representation of non-replaceable ending by A at the last 5–10 codons. Hence we conclude that it is the content of the wobble position of the codon, and not its fragility, that determines the spatial CUB pattern near the gene end.

### A-ending codons are associated with less stable mRNA folding at the gene 3’-end

Next, we wanted to test if the universal over-representation of A-ending codons at the 3’-end lower secondary RNA structures stability compared to what is expected from the general codon bias. To this end, we used RNAfold to compute minimum free energy (MFE) at the last 15 codons (observed MFE), and then replaced codons that end by A with the most frequently used codon for the corresponding amino acid in that species. If that codon also ends by A, we did not replace it. We computed the folding energy of the new sequence after replacing the last 15 codons (expected MFE). The results show (S10 Table) that in all species, the expected MFE was significantly lower than the observed MFE, indicating that the observed secondary structures at the 3’-end are less stable than the secondary structures expected from the general codon bias. These results suggest that the universal preference of A-ending codons at the 3’-end has a direct effect on lowering the stability of mRNA folding at that region.

### Highly expressed genes show stronger selection against GC-rich codons at the 3’-end

**Fig 8:**
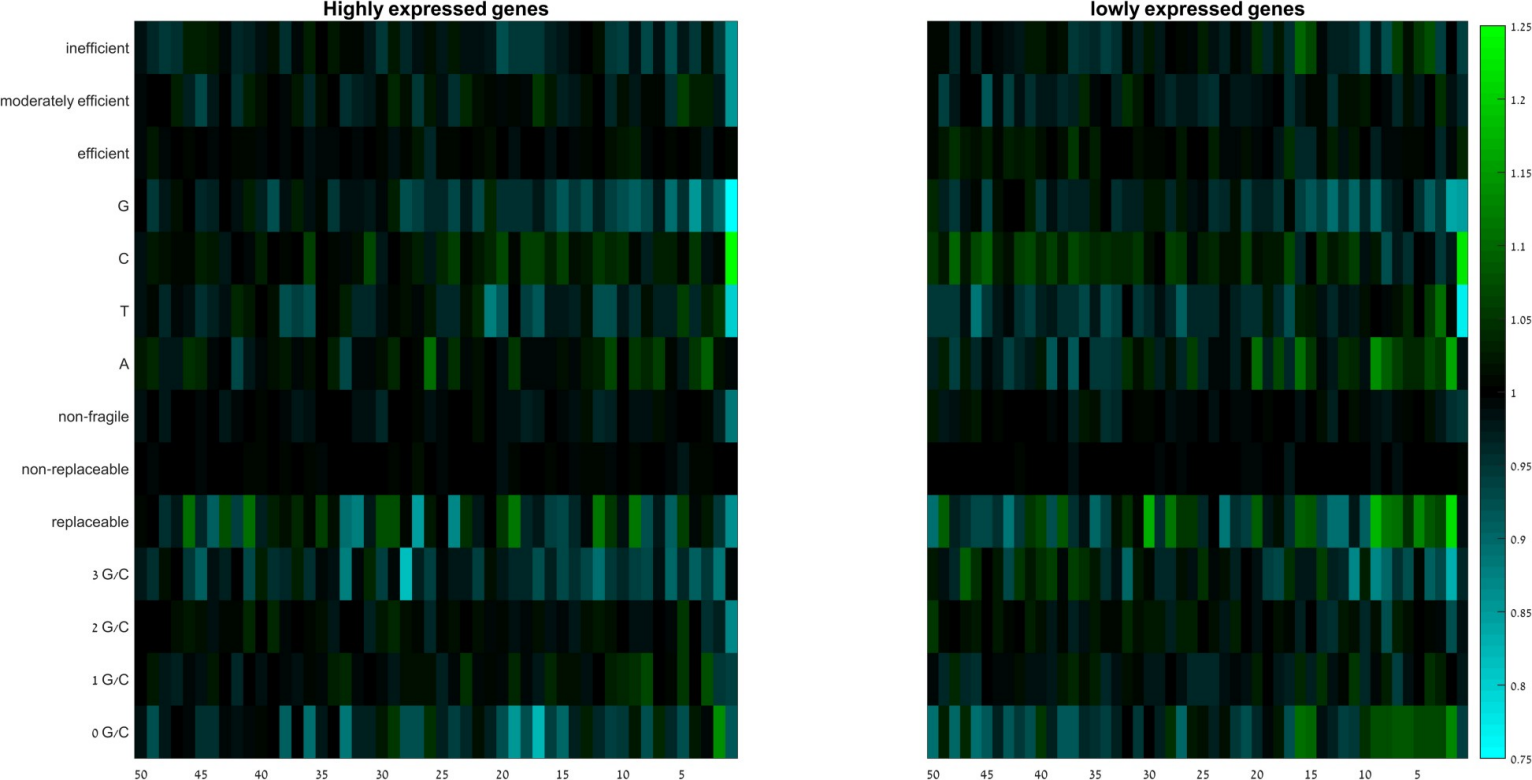
Group RSCA scores of all codons groups along the last 50 codons of human highly expressed and lowly expressed genes. Rows denote codons groups, columns denote positions. Species within domains are sorted from high (top) to low (bottom) GC-content.

It has been reported that highly expressed genes have stronger positional dependence of codon usage than lowly expressed genes in regions downstream of the 5’-end [47]. Given that highly expressed genes are assumed to be optimized for fast translation and low error rate, one may expect to see differences in positional CUB across genes with different expression levels. To check if this is the case at the 3’-end, we classified human genes to highly expressed and lowly expressed according to their average expression across 53 human tissues (see Methods; Fig 8). We found that the patterns of RSCA at the last 50 codons of highly expressed and lowly expressed genes behave similarly, and match the overall patterns reported above. There are nevertheless some minor differences. For example, strong depletion of G-ending codons starts earlier (at the 28^th^ codon) in highly expressed genes, while it starts later (at the 15^th^ codon) in lowly expressed genes. This is also the case for codons that are GC-rich (3/3 codons), where depletion starts earlier (at the 34^th^ codon) in highly expressed genes than in lowly expressed ones (at the 12^th^ codon). These results indicate that while the depletion of GC-rich and G-ending codons at the 3’-end is independent of gene expression, the strength of the depletion and the exact starting position might be affected by expression level. Interestingly, inefficient codons are more depleted toward the 3’-end in highly expressed genes, while lowly expressed genes show a mixture of patterns for these codons.

## Discussion

Codon usage bias near the gene start has received considerable attention, likely because of its assumed role in translation initiation [35, 47, 49]. In contrast, codon usage bias near the gene end gained less attention. Yet, codons in this region may affect translation termination, and therefore participate in translation regulation. Three main translation-related forces have been suggested to explain the unique nucleotide composition near the gene end, called in this work translation efficiency, translation accuracy, and mRNA folding. The main purpose of our work was to determine which of these three forces predominates codon usage patterns near the gene end.

Although several indices that measure codon usage bias have been developed, none directly accounts for the relative position of the codon along the gene. To fill this gap, we developed a measure called the relative spatial codon abundance (RSCA), which is a position-dependent index for codon usage bias. We focused at the 3’-end of the CDS, and used RSCA to evaluate the spatial CUB patterns of each codon in 91 species, covering all three domains of life. Grouping the codons according to various classification schemes, we were able to determine which of the three models above contribute most to the codon composition at the 3’-end of genes.

We noticed strike differences in codon usage patterns between eukaryotes and prokaryotes. Generally, C-ending and GC-rich codons are over-represented throughout most of the gene end in eukaryotes, whereas the opposite holds for A-ending, T-ending, and AT-rich codons. This observation cannot be attributed to elevated GC-content in eukaryotes, as the studies species from the three domains have similar GC content (*P* = 0.75, Kruskal-Wallis test; S2 Table). We suggest that the differences in spatial patterns that we see in this work likely reflect the high variability of the GC content along the coding region in eukaryotes, stemming from the fact that splicing sites and recombination hotspots are characterized by increased GC-content compared to other regions [50, 51]. The region roughly 100-200nt from each side of splicing sites is known to exhibit higher GC content compared to farther regions [51], which likely affects nucleotide composition around the TC. For example, in human, the median distance between the TC and the closest upstream splice site is 70nt. Moreover, it was found that codon usage is positively correlated with recombination rate (Marais & Piganeau, 2002), suggesting that Hill Robertson effect is a contributing factor affecting CUB. However, it is possible that this correlation is an artifact due to the mutational bias associated with recombination (Marais & Piganeau, 2002). For instance, since most optimal codons in *D*. *melanogaster* and *C*. *elegans* end in G or C, the enrichment of optimal codons observed at recombination hotspots in these species may be a byproduct of the mutational bias associated with high GC content in such regions (Marais, Mouchiroud, & Duret, 2001). Several studies suggest that GC-rich sequences are favored in gene conversion associated with recombination, an observation that was coined “biased gene conversion” (Bill, Duran, Miselis, & Nickoloff, 1998; Brown & Jiricny, 1988). It was claimed that this may explain the correlation between codon usage and recombination rate (Galtier, Piganeau, Mouchiroud, & Duret, 2001). Yet, weak effect of the Hill Robertson effect was found on codons usage after controlling for mutational bias, suggesting that it is a minor contributor to the observed codon usage bias (Marais & Piganeau, 2002). As a result, eukaryotes show high variability in the GC content along the coding region. Prokaryotes, lacking splicing and less affected by GC biased gene conversion and Hill Robertson effect, show lesser variability of the GC content within coding regions. In any case, RSCA is unaffected by overall biases in GC content but is sensitive to the changes in the codon usage across the sequence.

Interestingly, whereas A-ending codons also tend to be generally avoided at gene ends of eukaryotes, they are almost universally preferred along the last 10–15 codons (Fig 2), in agreement with previous studies [31, 36, 42]. However, our results show, for the first time, a variation in this pattern across kingdoms. For example, we show that C-ending codons are avoided near the gene end in prokaryotes, but in eukaryotes it is G-ending codons that are avoided. Moreover, the starting position of the switch in codons usage varies among species, which may reflect some lineage specific factors.

A notable exception to the universal preference of A-ending codons toward 3’-end is *C*. *elegans*. In C. *elegans* A-ending codons are significantly under-represented, while G-ending codons, and to a lesser extent T-ending ones, tend to be preferred. Yet AT-rich codons are still over-represented, while GC-rich codons are under-represented. The archaeon *N*. *martimus* deviates from all other species as it shows not only under-representation of A-ending and T-ending codons but also preference for GC-rich codons over AT-rich codons at the last 5 codons. A similar pattern can be seen in other AT-rich bacteria such as *C*. *jejuni*. Interestingly, Other AT-rich archaea such as M. *maripaludis*, in spite of showing neither over-representation nor under-representation of A-ending codons, still show preference to AT-rich and T-ending codons and avoidance of C-ending and GC-rich codons. Notably,most of the species that deviate from the general preference to A-ending and AT-rich codons near the gene end have small genomes, short genes, and are AT-rich. It is therefore possible that the general abundance of AT-rich codons makes the detection of 3’-end preference harder to detect. It is also possible that such genomes may have evolved different mechanisms to maintain the efficiency of translation termination. We noticed that these species nevertheless share the same trend toward enrichment in A-ending codons towards the gene end when introducing +1nt frameshift to the coding region (S5 and S7 Figs).

Selection for or against translation efficiency has been studied extensively and was shown to significantly contribute to codon usage bias at the gene start. At the gene end, Tuller *et al*., reported an enrichment in inefficient codons in some species, while others show the opposite trend [35]. There are two commonly used approaches to proxy a codon’s efficiency. The first computes the relative adaptiveness of a codon from the copy number of its cognate tRNA gene (the tAI measure), thus codons recognized by abundant tRNAs are considered more efficient than those recognized by rare tRNAs. The second computes the relative adaptiveness of a codon from its abundance in highly expressed genes (the CAI measure), thus codons that appear frequently in highly expressed gene are assigned higher efficiency. As the computation of relative adaptiveness from highly expressed genes is challenging in species with poor genomic annotations, and because the enrichment of specific codons in such genes may also be due to factors such as GC content [52], we preferred in this work to use the tAI index. Our work provides no support for appreciable contribution of translation efficiency to the codon composition near the gene end. The trends identified in previous reports disappear almost completely after controlling for the nucleotide content at the wobble position.

While selection against missense errors is believed to be independent on the position along the CDS [25], selection against nonsense errors has a strong positional dependence. It has been claimed that this is the dominant factor shaping spatial codon usage bias at the gene start and end in prokaryotes [25]. Some works make no clear distinction between translation efficiency and accuracy, and thus proxy the accuracy of a codon by its adaptiveness value, relying on the fact that inefficient codons are generally also more prone to translation errors. However, here we used a more direct approach. We looked at fragile codons, which are codons with higher likelihood to become a stop codon following a substitution [40]. We divided fragile codons to two groups: those that have no non-fragile synonymous codons (non-replaceable), and those that have at least one non-fragile synonymous codon (replaceable). We found that replaceable codons are generally over-represented along the last 10 codons in almost all species, while non-replaceable codons are neither over-represented nor under-represented in all species (Fig 6). This pattern may have three different explanations:

(1) Since truncation of the protein may be less harmful toward its very end, there might be a relief in the strength of selection against replaceable codons towards the CDS end.

(2) Replaceable codons are enriched for codons that end with A (71% of the replaceable codons, but only 27% of the non-replaceable and 14% of the non-fragile; S2 Table), thus their enrichment or depletion towards the gene end are largely dictated by the nucleotide composition of the wobble position.

(3) Replaceable codons are enriched in translationally inefficient codons. On average, 20–25% of inefficient codons at the species of every domain are also replaceable, compared to 10% of moderately efficient codons and 5% of efficient codons. Therefore, their enrichment or depletion towards the gene end are largely dictated by the level of codon efficiency.

Our results rule out the third possibility, as we have shown that codon efficiency does not contribute significantly to the spatial CUB near the gene end. Our results also rule out the first explanation, as codons ending with different nucleotides show different spatial patterns (Fig 7, S12–S14 Figs; S4, S5, S8 and S9 Tables). We therefore concluded that it is the content of the wobble position that predominantly determines the spatial CUB at the gene end. Thus, neither selection against nonsense errors nor its relief at the far end of the gene are major contributors to the spatial codon usage bias at the gene 3’-end.

If the nucleotide composition is the main contributor to the codon usage bias at the 3’-end, we expect to see variability in the strength of this bias between highly and lowly expressed genes. Hockeberry *et al*. [47] studied the codon usage bias in *E*.*coli* from a positional context, and found that highly and lowly expressed genes behave similarly at the 5’-region, but positional dependency was significantly higher for highly expressed genes at distal parts. Part of the positional dependency was related to the increased usage of A/T rich codons just after the gene start, and part of it, in particular the 4- and 6-fold degenerate codons, was related to gene copy number of cognate tRNA. Because expression data for human is available for many different tissues, we used human as a model to study the effect of gene expression on the RSCA pattern at the 3’-end (Fig 8). Our results show that the avoidance of GC-rich and G-ending codons starts earlier in highly expressed genes and the strength of the depletion is much stronger at the last 5 codons. This suggests that the strength of selection pressure against secondary structures toward the 3’-end depends on the gene expression level. Interestingly, our results also show a strong under-representation of inefficient codons near the 3’-end in highly expressed genes. Highly expressed genes avoid the usage of inefficient codons to maintain proper translation speed specifically at the 5’-end [49]. Our results show that this avoidance is present near the 3’-end.

To sum up, we conclude that neither selection against translation efficiency, nor selection for translation accuracy have a significant role in shaping the spatial codon usage bias at the gene 3’-end. Our work revealed that what mainly governs this pattern is the codon nucleotide content, especially at its wobble position. We showed that A/T-ending codons are preferred at the gene end, while C/G-ending codons are avoided. We have shown this using species from all three domains, which cover a wide range of GC contents and gene lengths (S1 Table). Thus, it is unlikely that species-specific factors such as particular evolutionary history, genome size, and Hill-Robertson effect can explain this observation. Instead, our results are fully compatible with the notion that codon usage patterns towards the gene end are generally a result of selective forces against the formation of RNA secondary structures near the stop codon [31]. It has been shown that GC-rich codons are associated with more stable RNA secondary structures [53]. We showed here that the preference toward A-ending codons lower the stability of mRNA folding at the 3’-end (S10 Table). The presence of mRNA secondary structures along the CDS stalls ribosome elongation [54], and lead to translation abortion [31, 55]. Moreover, translation termination requires the recruitment of release factors RF1, RF2, and RF3. Aberrant, and especially delayed, kinetics of the interactions of these release factors with the stalling ribosome may trigger translation read-through, whereby a near cognate tRNA recognizes the stop codon as a sense codon [31, 56]. We therefore suggest that there is a strong selection against the formation of mRNA secondary structures at the vicinity of the stop codon in order to allow for normal translation termination. We have shown that this nucleotide composition signature is also apparent at the 3’UTR side of the stop codon [57].

## Methods

### RSCA computation

Let *G* be a set of genes, and let *l*_*g*_ be the length, measured by the number of codons, of gene *g* in the set. We are interested in computing the spatial distribution of codons along the last Δ*L* codons, and will hereinafter assume that all genes in the set *G* are of minimal length Δ*L*. Let *C* = Σ_*g*∈*G*_*l*_*g*_ be the total number of codons in our gene set, and let $N_{l}=\Sigma_{g\in G}1_{\left\{l_{g}\geq l\right\}}$ be the number of genes whose length is at least *l*.

Let the position of a codon, *α*, be its consecutive number from the gene end. For example the, last codon of a gene has *α* = 1. Let $c_{g\alpha }^{i}$ be 1 if codon type *i* is at position *α* in gene *g*, and 0 otherwise. For example, $c_{g\alpha }^{CUU}=1$ means that codon CUU is present at position *α* in gene *g*. If a position is not present in a gene, for example if we are looking at position 10 for a gene whose length is 9, we set $c_{g\alpha }^{i}$ to zero for all *i*. Let $C^{i}=\Sigma_{g\in G}\Sigma_{\alpha =1}^{l_{g}}c_{g\alpha }^{i}$ be the total number of times that codon of type *i* appears in our gene set. Similarly, let $C_{\alpha }^{i}=\Sigma_{g\in G}c_{g\alpha }^{i}$ be the total number of codons of type *i* at position *α* in our gene set. The total frequency of codon type *i* is *p*^*i*^ = *C*^*i*^/*C* = *C*^*i*^/∑_*i*_*C*^*i*^. Assuming uniform distribution of each codon both within and between the genes in *G*, the expected number of times that codon type *i* would appear at position *α* in our gene set is $E_{\alpha }^{i}=p^{i}\cdot N_{\alpha }$ We define the *spatial codon abundance* of codon *i* at position *α* as the ratio of the observed to the expected number

$$Q_{\alpha }^{i}=\frac{C_{\alpha }^{i}}{E_{\alpha }^{i}}.$$

$Q_{\alpha }^{i}=1$ describes a codon whose frequency at position *α* if exactly as expected if it were uniformly distributed along the gene. $Q_{\alpha }^{i}>1$ describes a codon that is over-represented at position *α*, whereas $Q_{\alpha }^{i}<1$ describes a codon that is under-represented at position *α*. To measure how significantly $Q_{\alpha }^{i}$ deviates from one, we compute its standard error. Since $E_{\alpha }^{i}$ is a binomial random variable, its standard deviation is $\Delta E_{\alpha }^{i}=\sqrt{p^{i}(1-p^{i})N_{\alpha }}=\sqrt{(1-p^{i})E_{\alpha }^{i}}$. By means of error propagation, the standard error of $Q_{\alpha }^{i}$ is

$$\Delta Q_{\alpha }^{i}=Q_{\alpha }^{i}\cdot \frac{\Delta E_{\alpha }^{i}}{E_{\alpha }^{i}}=Q_{\alpha }^{i}\sqrt{\frac{1-p^{i}}{E_{\alpha }^{i}}}.$$

$Q_{\alpha }^{i}$ allows the measurement of codon usage bias towards the gene end. However, this bias is built from two contributions: the bias of the coded amino acid, and the bias of the codon itself. For example, assume that the UGG codon is over-represented towards the gene end. However, since it is the only codon for tryptophan, this bias may be entirely due to an over-representation of tryptophan at the gene end. In this work, we are interested only in the contribution of the codon itself to the bias. To take this into account, let *A*(*i*) be the set of codons that are synonymous to codon type *i* (including *i* itself). Let $A_{\alpha }^{i}=\Sigma_{j\in A(i)}\sum_{g\in G}c_{g\alpha }^{j}$ be the total number of times any codon from the set *A*(*i*) appears at position *α* in our gene set. Let us define the relative usage of codon *i* as *f*^*i*^ = *C*^*i*^/Σ_*j*∈*A*(*i*)_*C*^*j*^. Then, the expected number of times that codon *i* will be used at position *α* is $H_{\alpha }^{i}=f^{i}A_{\alpha }^{i}$. We define the *relative spatial codon abundance* (RSCA) of codon *i* at position *α*, after accounting for the amino acid spatial bias, as

$$R_{\alpha }^{i}=\frac{C_{\alpha }^{i}}{H_{\alpha }^{i}}.$$

This is the value that we use throughout this work. Since $H_{\alpha }^{i}$ is a binomial random variable, its standard deviation is $\Delta H_{\alpha }^{i}=\sqrt{f_{i}(1-f_{i})A_{\alpha }^{i}}=\sqrt{(1-f_{i})H_{\alpha }^{i}}$. By means of error propagation, the standard error of $R_{\alpha }^{i}$ is

$$\Delta R_{\alpha }^{i}=R_{\alpha }^{i}\cdot \frac{\Delta H_{\alpha }^{i}}{H_{\alpha }^{i}}=R_{\alpha }^{i}\sqrt{\frac{1-f_{i}}{H_{\alpha }^{i}}}.$$

With this measure, the codon UGG from the example above would have $R_{\alpha }^{UGG}=1$ for all *α*, as all the bias is entirely explained by the amino acid.

### Group RSCA score

Whereas $R_{\alpha }^{i}$ measures the spatial abundance of a certain codon, in this work we will mostly be interested in the spatial patterns characterizing a group of *n* codons, *S*. We therefore define the group RSCA, $R_{\alpha }^{S}$, as the median of $R_{\alpha }^{i}$ scores of *S* codons which we used in the entire work.

### Genomic data

Gene coding sequences of the studied species were downloaded from Ensembl [58]. We randomly sampled half of the eukaryotic species from the ensemble eukaryotic collection at ftp://ftp.ensembl.org/pub/currentfasta. For bacteria and archaea, we used the Enesmbl bacterial collection “bacterial 0 collection”. We again randomly sampled half of the species. However, some species, such as *Buchnera aphidicola*, produce many missing values. We filtered out such species, ending up with 34 eukaryotes, 49 bacteria (in which 3 utilize Mycoplasma genetic code), and 8 archaea. If a gene had multiple isoforms, we included only the canonical form (longest CDS) in our analyses. We removed genes whose coding sequence was shorter than 100 codons, as well as genes whose annotation included obvious mistakes such as total coding length not divisible by three, lack of a stop codon, or the presence of internal stop codons.

### Relative adaptiveness value

We computed relative adaptiveness values following the derivation of dos Reis *et al*. [7]. For codon type *i*, the absolute adaptiveness value is

$$W_{i}=\sum_{j=1}^{n_{i}}(1-s_{ij})C_{ij},$$

where *n*_*i*_ is the number of tRNAs that recognize codon type *i*, *s*_*ij*_ is a selective constraint of the coupling efficiency between the *j*th tRNA and the *i*th codon type, and *C*_*ij*_ is the copy number of the *j*th tRNA that recognizes the codon *i*. The relative adaptiveness value is defined as

$$w_{i}=\left\{ \begin{array}{cc} W_{i}/W_{max} & W_{i}\neq 0\\ W_{0} & W_{i}=0 \end{array}\right.,$$

where *W*_0_ is the geometric mean of all the *W*_*i*_ values.

Relative adaptiveness value was computed for all codons of each species after retrieving the corresponding tRNA genes copy numbers from genomic tRNA database http://lowelab.ucsc.edu/GtRNAdb. Based on these values, we binned the codons in each species into three groups: Codons with relative adaptiveness at the bottom third are denote inefficient codons; codons with relative adaptiveness at the middle third are denote moderate-efficient codons; and codons with relative adaptiveness at the upper third are denote efficient codons.

### Classifying human genes to highly and lowly expressed genes

We obtained from Genotype-Tissue Expression (GTEX) project [59], the expression of 57,073 genes from 53 human tissues. We computed the average expression of each gene in all the tissues, and filtered out genes that have very low expression (less than 0.5) and those that lack coding region. The 15,471 remaining genes were classified as ‘highly expressed genes’ if their average expression is higher than the 75th percentile (3,817 genes) and as ‘lowly expressed genes’ if their average expression is below the 25th percentile (3,817 genes).

## Supporting information

S1 FigGroup RSCA scores ($R_{\alpha }^{S}$) of efficient, moderately efficient, and inefficient codons along the last 50 codons of the gene for random codon permutation.Rows denote species, columns denote positions.(PDF)

S2 FigGroup RSCA scores ($R_{\alpha }^{S}$) of A-ending, T-ending, C-ending, and G-ending codons along the last 50 codons of the gene for random codon permutation.Rows denote species, columns denote positions.(PDF)

S3 FigGroup RSCA scores ($R_{\alpha }^{S}$) of replaceable, non-replaceable, and non-fragile codons along the last 50 codons of the gene for random codon permutation.Rows denote species, columns denote positions.(PDF)

S4 FigGroup RSCA scores ($R_{\alpha }^{S}$) of 0, 1, 2, and 3 G/C codons along the last 50 codons of the gene for random codon perumtation.Rows denote species, columns denote positions.(PDF)

S5 FigGroup RSCA scores ($R_{\alpha }^{S}$) of A-ending, T-ending, C-ending, and G-ending triplets after introducing +1nt frameshift to the coding region (i.e., examining the first codon position in the original coding sequence) along the last 50 codons of the gene.Rows denote species, columns denote positions. Species within domains are sorted from high (top) to low (bottom) GC-content.(PDF)

S6 FigGroup RSCA scores ($R_{\alpha }^{S}$) of A-ending, T-ending, C-ending, and G-ending codons after introducing +2nt frameshift to the coding region (i.e., examining the second codon position in the original coding sequence) along the last 50 codons of the gene.Rows denote species, columns denote positions. Species within domains are sorted from high (top) to low (bottom) GC-content.(PDF)

S7 FigGroup RSCA scores ($R_{\alpha }^{S}$) of 0, 1, 2, and 3 G/C nucleotides in codons after introducing +1nt frameshift to the coding region (i.e., examining the first codon position in the original coding sequence) along the last 50 codons of the gene.Rows denote species, columns denote positions. Species within domains are sorted from high (top) to low (bottom) GC-content.(PDF)

S8 FigGroup RSCA scores ($R_{\alpha }^{S}$) of 0, 1, 2, and 3 G/C nucleotides in codons after introducing +2nt frameshift to the coding region (i.e., examining the second codon position in the original coding sequence) along the last 50 codons of the gene.Rows denote species, columns denote positions. Species within domains are sorted from high (top) to low (bottom) GC-content.(PDF)

S9 FigGroup RSCA scores ($R_{\alpha }^{S}$) of efficient, moderately efficient, and inefficient codons ending by T along the last 50 codons of the gene.Rows denote species, columns denote positions. Species where none of the codons in a particular efficiency group end by T (missing data) are shown as white stripes. Species within domains are sorted from high (top) to low (bottom) GC-content.(PDF)

S10 FigGroup RSCA scores ($R_{\alpha }^{S}$) of efficient, moderately efficient, and inefficient codons ending by C along the last 50 codons of the gene.Rows denote species, columns denote positions. Species where none of the codons in a particular efficiency group end by C (missing data) are shown as white stripes. Species within domains are sorted as in S9 Fig.(PDF)

S11 FigGroup RSCA scores ($R_{\alpha }^{S}$) of efficient, moderately efficient, and inefficient codons ending by G along the last 50 codons of the gene.Rows denote species, columns denote positions. Species where none of the codons in a particular efficiency group end by G (missing data) are shown as white stripes. Species within domains are sorted as in S9 Fig.(PDF)

S12 FigGroup RSCA scores ($R_{\alpha }^{S}$) of replaceable, non-replaceable, and non-fragile codons ending by T along the last 50 codons of the gene.Rows denote species, columns denote positions. As there are no replaceable codons that end by T, all species are depicted by white stripes (missing data). Species within domains are sorted from high (top) to low (bottom) GC-content.(PDF)

S13 FigGroup RSCA scores ($R_{\alpha }^{S}$) of replaceable, non-replaceable, and non-fragile codons ending by C along the last 50 codons of the gene.Rows denote species, columns denote positions. As there are no replaceable codons that end by C, all species are depicted by white stripes (missing data). Species within domains are sorted as in S12 Fig.(PDF)

S14 FigGroup RSCA scores ($R_{\alpha }^{S}$) of replaceable, non-replaceable, and non-fragile codons ending by G along the last 50 codons of the gene.Rows denote species, columns denote positions. Species within domains are sorted as in S12 Fig.(PDF)

S1 Table List of the species analyzed, and some properties of their genes. (PDF)

S2 Table For each species and for every pair of codon classification scheme where efficiency is part of, we computed the (FDR corrected) p-value of the χ2 independence test. (PDF)

S3 Table Linear regression of group RSCA score at the last 10, 20, and 30 codons, for spatially randomized codons. Data for each species is shown in a different excel sheet. (XLSX)

S4 Table Linear regression of group RSCA score at the last 10, 20, and 30 codons. Data for each species is shown in a different excel sheet.
(XLSX)

S5 Table Linear model scores for each codon group and species. The value 1 indicates a significant increase toward the 3’-end in the last 10, 20, or 30 codons. The value -1 indicates a significant decrease, and 0 indicates insignificant linear fit.
(XLSX)

S6 Table For each position (distance from the stop codon, columns) and for every species, we computed the Kruskal-Wallis (FDR corrected) p-value, measuring the difference in the distribution of RSCA values between efficient, inefficient, and moderately efficient that end by A/T. (PDF)

S7 Table For each position (distance from the stop codon, columns) and for every species, we computed the Kruskal-Wallis (FDR corrected) p-value, measuring the difference in the distribution of RSCA values between efficient, inefficient, and moderately efficient codons that end by C/G. (PDF)

S8 Table For each position (distance from the stop codon, columns) and for every species, we computed the Kruskal-Wallis (FDR corrected) p-value, measuring the difference in the distribution of RSCA values between replaceable, non-replaceable, and non-fragile that end by A/T. (PDF)

S9 Table For each position (distance from the stop codon, columns) and for every species, we computed the Kruskal-Wallis (FDR corrected) p-value, measuring the difference in the distribution of RSCA values between replaceable, non-replaceable, and non-fragile codons that end by C/G. (PDF)

S10 Table Median minimum free energy (MFE) at the 3’-end (observed MFE), median MFE expected by the global (not spatial) codon bias (expected MFE), and p-value for testing whether the median difference between pairs of expected and observed values equals zero (FDR-corrected two-sided sign test). (PDF)
