# Supplementary material for: Nucleotide composition affects codon usage toward the 3'-end
Source: PLoS One. 2019 Dec 4;14(12):e0225633. doi: 10.1371/journal.pone.0225633 (PMC6892556; doi:10.1371/journal.pone.0225633)
Supplement: S2 Fig — Rows denote species, columns denote positions. (PDF) [file pone.0225633.s002.pdf]

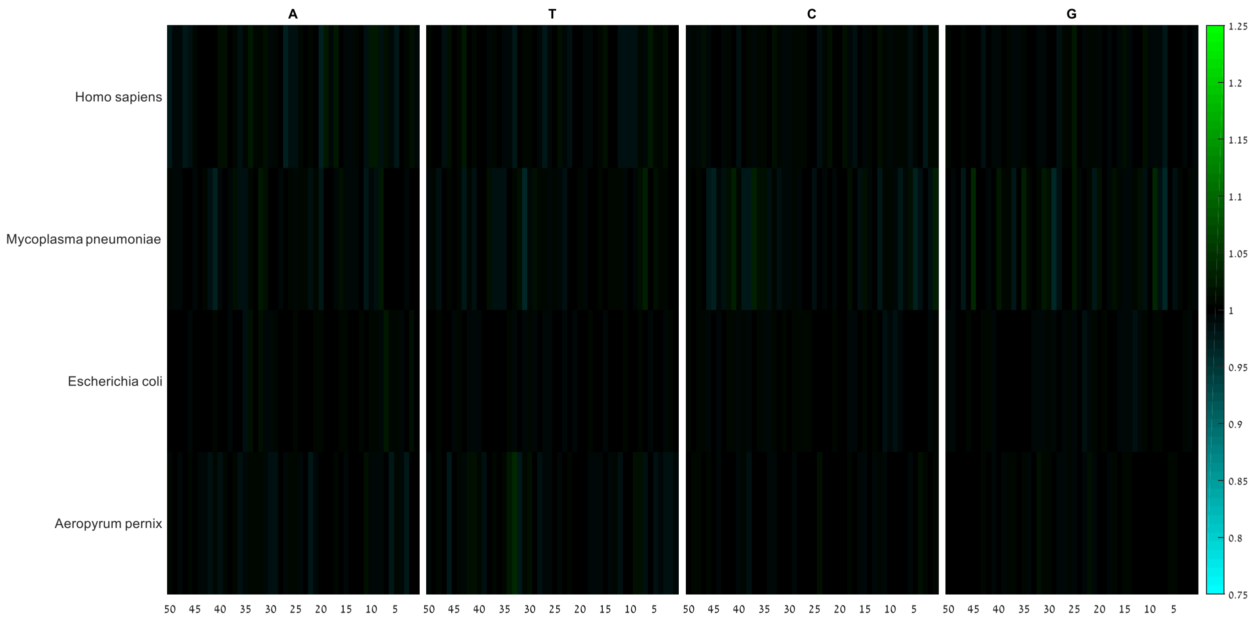

Figure S2. Group RSCA scores (R_{α}^{S}) of A-ending, T-ending, C-ending, and G-ending codons along the last 50 codons of the gene for random codon permutation. Rows denote species, columns denote positions.
