# Supplementary material for: Nucleotide composition affects codon usage toward the 3'-end
Source: PLoS One. 2019 Dec 4;14(12):e0225633. doi: 10.1371/journal.pone.0225633 (PMC6892556; doi:10.1371/journal.pone.0225633)
Supplement: S3 Fig — Rows denote species, columns denote positions. (PDF) [file pone.0225633.s003.pdf]

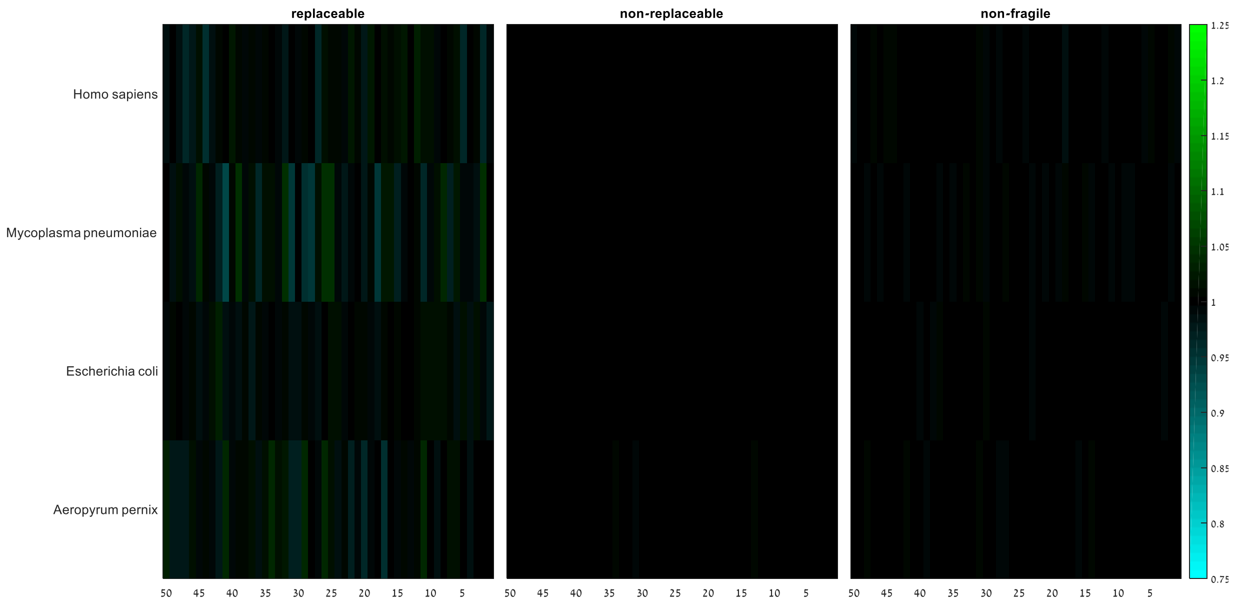

Figure S3. Group RSCA scores (R_α^S) of replaceable, non-replaceable, and non-fragile codons along the last 50 codons of the gene for random codon permutation. Rows denote species, columns denote positions.
