# Supplementary material for: Nucleotide composition affects codon usage toward the 3'-end
Source: PLoS One. 2019 Dec 4;14(12):e0225633. doi: 10.1371/journal.pone.0225633 (PMC6892556; doi:10.1371/journal.pone.0225633)
Supplement: S7 Fig — Rows denote species, columns denote positions. Species within domains are sorted from high (top) to low (bottom) GC-content. (PDF) [file pone.0225633.s007.pdf]

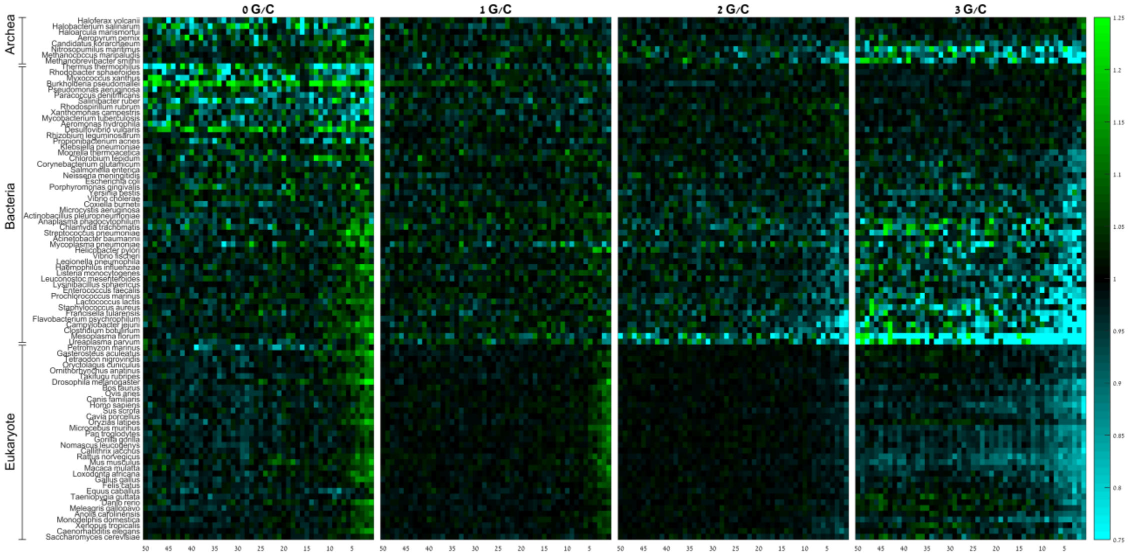


**Figure S7. Group RSCA scores ($R_{\alpha}^{S}$) of 0, 1, 2, and 3 G/C nucleotides in codons after introducing +1nt frameshift to the coding region (i.e., examining the first codon position in the original coding sequence) along the last 50 codons of the gene**. Rows denote species, columns denote positions. Species within domains are sorted from high (top) to low (bottom) GC-content.
