# Supplementary material for: Nucleotide composition affects codon usage toward the 3'-end
Source: PLoS One. 2019 Dec 4;14(12):e0225633. doi: 10.1371/journal.pone.0225633 (PMC6892556; doi:10.1371/journal.pone.0225633)
Supplement: S8 Fig — Rows denote species, columns denote positions. Species within domains are sorted from high (top) to low (bottom) GC-content. (PDF) [file pone.0225633.s008.pdf]

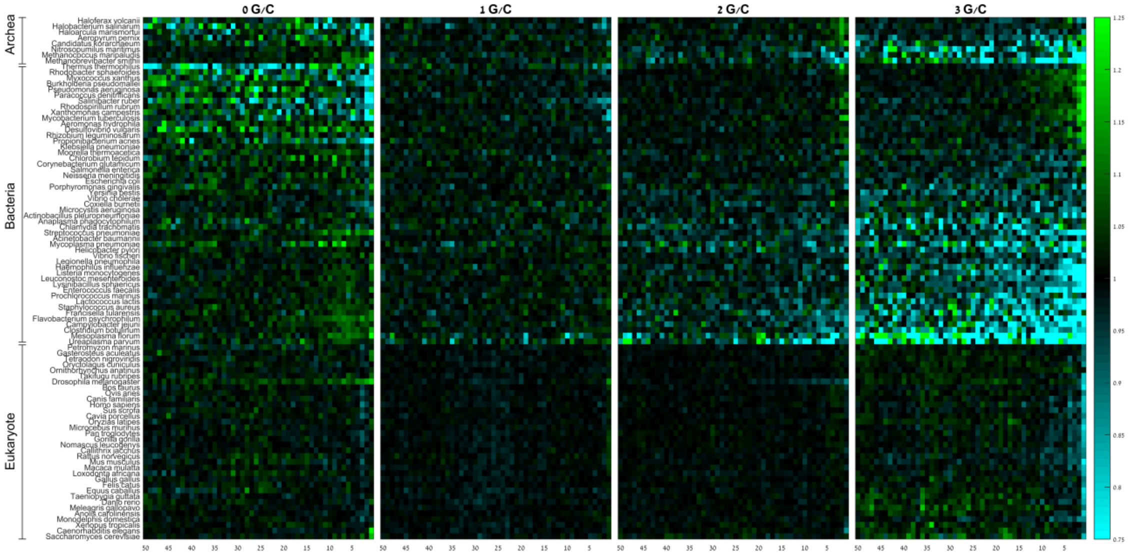

Figure S8. Group RSCA scores (R^{S}_{α}) of 0, 1, 2, and 3 G/C nucleotides in codons after introducing +2nt frameshift to the coding region (i.e., examining the second codon position in the original coding sequence) along the last 50 codons of the gene. Rows denote species, columns denote positions. Species within domains are sorted from high (top) to low (bottom) GC-content.
