# Supplementary material for: Nucleotide composition affects codon usage toward the 3'-end
Source: PLoS One. 2019 Dec 4;14(12):e0225633. doi: 10.1371/journal.pone.0225633 (PMC6892556; doi:10.1371/journal.pone.0225633)
Supplement: S11 Fig — Rows denote species, columns denote positions. Species where none of the codons in a particular efficiency group end by G (missing data) are shown as white stripes. Species within domains are sorted as in S9 Fig. (PDF) [file pone.0225633.s011.pdf]

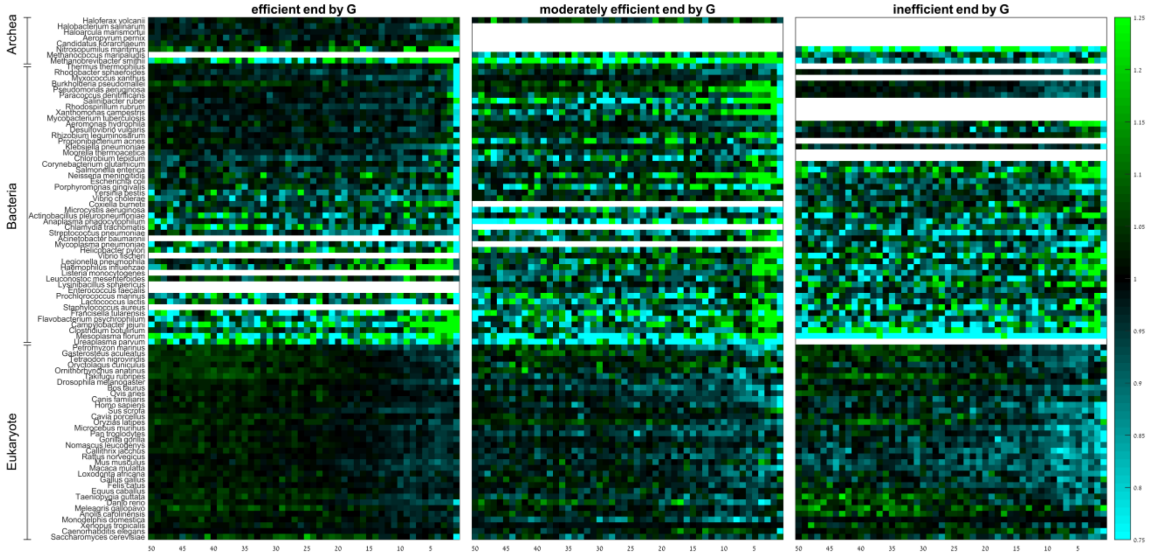

Figure S11. Group RSCA scores (R_{α}^{S}) of efficient, moderately efficient, and inefficient codons ending by G along the last 50 codons of the gene. Rows denote species, columns denote positions. Species where none of the codons in a particular efficiency group end by G (missing data) are shown as white stripes. Species within domains are sorted as in Figure S9.
