# Supplementary material for: Nucleotide composition affects codon usage toward the 3'-end
Source: PLoS One. 2019 Dec 4;14(12):e0225633. doi: 10.1371/journal.pone.0225633 (PMC6892556; doi:10.1371/journal.pone.0225633)
Supplement: S12 Fig — Rows denote species, columns denote positions. As there are no replaceable codons that end by T, all species are depicted by white stripes (missing data). Species within domains are sorted from high (top) to low (bottom) GC-content. (PDF) [file pone.0225633.s012.pdf]

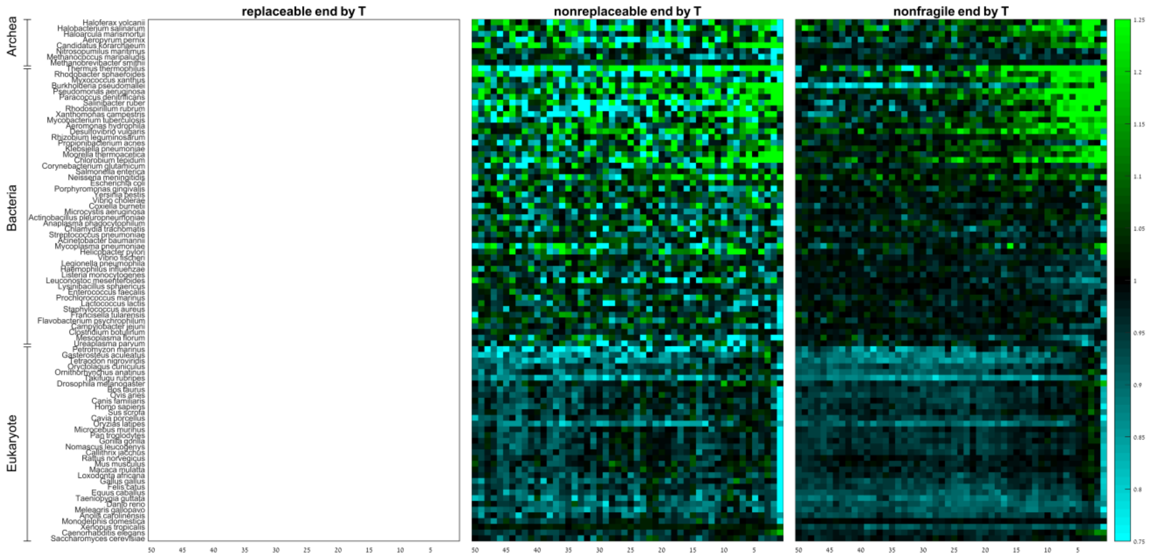

Figure S12. Group RSCA scores (R_{α}^{S}) of replaceable, non-replaceable, and non-fragile codons ending by T along the last 50 codons of the gene. Rows denote species, columns denote positions. As there are no replaceable codons that end by T, all species are depicted by white stripes (missing data). Species within domains are sorted from high (top) to low (bottom) GC-content.
