# Supplementary material for: Nucleotide composition affects codon usage toward the 3'-end
Source: PLoS One. 2019 Dec 4;14(12):e0225633. doi: 10.1371/journal.pone.0225633 (PMC6892556; doi:10.1371/journal.pone.0225633)
Supplement: S14 Fig — Rows denote species, columns denote positions. Species within domains are sorted as in S12 Fig. (PDF) [file pone.0225633.s014.pdf]

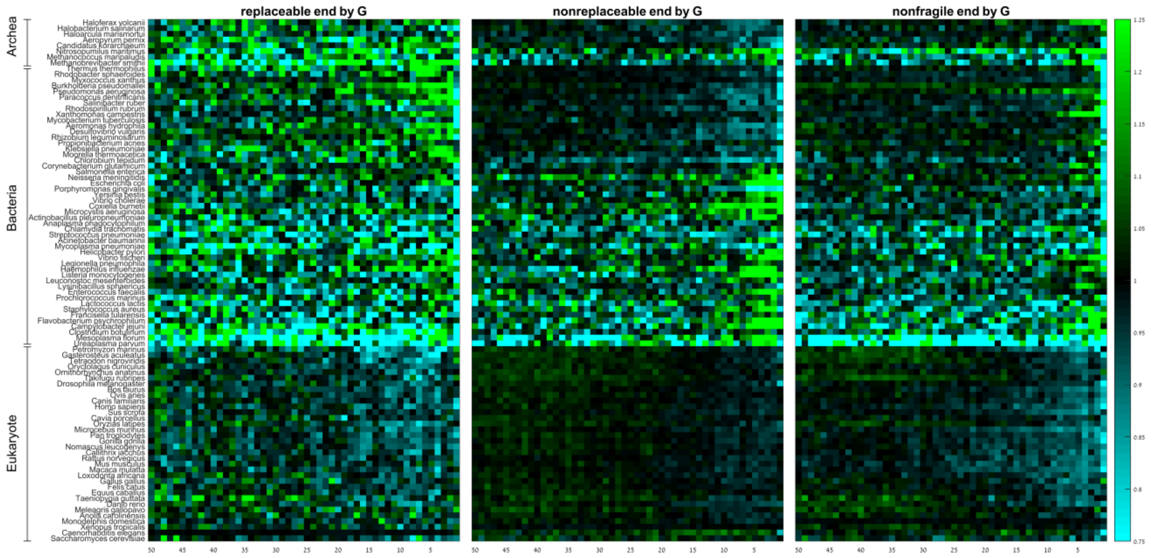

Figure S14. Group RSCA scores (R^S_α) of replaceable, non-replaceable, and non-fragile codons ending by G along the last 50 codons of the gene. Rows denote species, columns denote positions. Species within domains are sorted as in Figure S12.
