# Supplementary material for: Nucleotide composition affects codon usage toward the 3'-end
Source: PLoS One. 2019 Dec 4;14(12):e0225633. doi: 10.1371/journal.pone.0225633 (PMC6892556; doi:10.1371/journal.pone.0225633)
Supplement: S1 Table — (PDF) [file pone.0225633.s015.pdf]

Species	Number of genes	Median gene length	A content	C content	G content	T content
Anolis carolinensis	18553	1200	0.28	0.23	0.25	0.23
Bos taurus	19978	1167	0.24	0.27	0.27	0.21
Caenorhabditis elegans	20320	993	0.30	0.21	0.22	0.27
Callithrix jacchus	20933	1134	0.26	0.26	0.26	0.22
Canis familiaris	19847	1206	0.25	0.26	0.27	0.21
Cavia porcellus	18640	1173	0.25	0.26	0.27	0.22
Danio rerio	25659	1206	0.27	0.24	0.25	0.23
Drosophila melanogaster	13893	1182	0.25	0.27	0.27	0.21
Equus caballus	20427	1092	0.26	0.26	0.26	0.22
Felis catus	20427	1092	0.26	0.26	0.26	0.22
Gallus gallus	20427	1092	0.26	0.26	0.26	0.22
Gasterosteus aculeatus	20784	1149	0.24	0.28	0.28	0.20
Gorilla gorilla	20699	1131	0.25	0.26	0.26	0.22
Homo sapiens	22680	1233	0.25	0.27	0.27	0.22
Loxodonta africana	20023	1107	0.25	0.26	0.26	0.22
Macaca mulatta	21037	1122	0.26	0.25	0.26	0.22
Meleagris gallopavo	14104	1188	0.28	0.23	0.25	0.23
Microcebus murinus	18071	1224	0.25	0.26	0.27	0.22
Monodelphis domestica	21307	1167	0.27	0.24	0.24	0.24
Mus musculus	22606	1137	0.25	0.26	0.26	0.22
Nomascus leucogenys	18569	1221	0.25	0.26	0.26	0.22
Ornithorhynchus anatinus	21674	825	0.24	0.27	0.27	0.21
Oryctolagus cuniculus	19284	1161	0.24	0.27	0.27	0.21
Oryzias latipes	19662	1125	0.25	0.26	0.27	0.22
Ovis aries	20882	1128	0.25	0.26	0.27	0.21

Pan troglodytes	18688	1212	0.25	0.26	0.26	0.22
Petromyzon marinus	10280	894	0.21	0.30	0.30	0.18
Rattus norvegicus	22255	1107	0.25	0.26	0.26	0.22
Saccharomyces cerevisiae	6650	1079	0.32	0.19	0.21	0.28
Sus scrofa	21600	1014	0.25	0.26	0.27	0.22
Taeniopygia guttata	17454	948	0.26	0.25	0.26	0.22
Takifugu rubripes	18514	1287	0.25	0.27	0.27	0.21
Tetraodon nigroviridis	19593	1145	0.24	0.28	0.28	0.20
Xenopus tropicalis	18442	1227	0.29	0.22	0.24	0.25
Acinetobacter baumannii	7420	813	0.30	0.19	0.21	0.29
Actinobacillus pleuropneumoniae	2012	819	0.30	0.19	0.23	0.27
Aeromonas hydrophila	4106	870	0.19	0.32	0.31	0.18
Anaplasma phagocytophilum	1303	593	0.28	0.17	0.26	0.29
Burkholderia pseudomallei	6320	981	0.16	0.34	0.35	0.15
Campylobacter jejuni	1619	813	0.37	0.13	0.18	0.32
Chlamydia trachomatis	894	870	0.28	0.19	0.22	0.30
Chlorobium tepidum	2216	720	0.22	0.28	0.28	0.20
Clostridium botulinum	3401	780	0.41	0.11	0.18	0.30
Corynebacterium glutamicum	3052	810	0.23	0.27	0.27	0.23
Coxiella burnetii	1823	738	0.29	0.20	0.23	0.28
Desulfovibrio vulgaris	3496	777	0.19	0.32	0.30	0.18
Enterococcus faecalis	3241	750	0.34	0.17	0.21	0.28
Escherichia coli	4099	849	0.24	0.24	0.27	0.24

Flavobacterium psychrophilum	2443	846	0.38	0.15	0.17	0.29
Francisella tularensis	1604	786	0.36	0.14	0.19	0.31
Haemophilus influenzae	1707	789	0.32	0.18	0.21	0.29
Helicobacter pylori	1541	801	0.32	0.18	0.22	0.28
Klebsiella pneumoniae	5169	843	0.21	0.28	0.30	0.20
Lactococcus lactis	2264	756	0.34	0.16	0.20	0.30
Legionella pneumophila	3164	861	0.32	0.18	0.21	0.29
Leuconostoc mesenteroides	1998	804	0.32	0.17	0.21	0.29
Listeria monocytogenes	2845	804	0.34	0.17	0.21	0.28
Lysinibacillus sphaericus	4771	699	0.33	0.16	0.21	0.29
Mesoplasma florum	683	891	0.41	0.11	0.16	0.32
Microcystis aeruginosa	6308	555	0.29	0.20	0.22	0.28
Moorella thermoacetica	2463	813	0.22	0.28	0.29	0.21
Mycobacterium tuberculosis	4015	846	0.17	0.32	0.33	0.17
Mycoplasma pneumoniae	688	857	0.32	0.19	0.20	0.28
Myxococcus xanthus	7306	918	0.15	0.34	0.35	0.15
Neisseria meningitidis	1906	762	0.25	0.27	0.26	0.21
Paracoccus denitrificans	5077	837	0.16	0.33	0.34	0.16
Porphyromonas gingivalis	1892	882	0.26	0.24	0.25	0.24
Prochlorococcus marinus	1879	699	0.33	0.16	0.21	0.30
Propionibacterium acnes	2297	873	0.19	0.30	0.30	0.21

Pseudomonas aeruginosa	5899	822	0.17	0.35	0.32	0.16
Rhizobium leguminosarum	7131	855	0.19	0.31	0.30	0.19
Rhodobacter sphaeroides	4277	822	0.15	0.34	0.34	0.16
Rhodospirillum rubrum	3839	888	0.16	0.34	0.32	0.18
Salinibacter ruber	2827	912	0.17	0.33	0.33	0.16
Salmonella enterica	4542	816	0.24	0.25	0.28	0.23
Staphylococcus aureus	2614	776	0.36	0.14	0.19	0.30
Streptococcus pneumoniae	2085	723	0.30	0.18	0.22	0.29
Thermus thermophilus	2230	777	0.15	0.35	0.35	0.15
Ureaplasma parvum	611	864	0.40	0.12	0.14	0.34
Vibrio cholerae	3771	780	0.26	0.23	0.25	0.26
Vibrio fischeri	3799	855	0.31	0.18	0.21	0.30
Xanthomonas campestris	4173	876	0.17	0.33	0.33	0.17
Yersinia pestis	4188	789	0.26	0.23	0.26	0.26
Aeropyrum pernix	1697	767	0.23	0.24	0.33	0.20
Candidatus korarchaeum	1602	789	0.27	0.20	0.30	0.23
Haloarcula marismortui	4225	728	0.21	0.31	0.31	0.17
Halobacterium salinarum	2702	726	0.18	0.33	0.33	0.15
Haloferax volcanii	4018	759	0.18	0.35	0.32	0.15
Methanobrevibacter smithii	1795	750	0.37	0.13	0.19	0.31
Methanococcus maripaludis	1721	756	0.38	0.14	0.19	0.28
Nitrosopumilus maritimus	1795	678	0.37	0.15	0.19	0.28

Table S1. List of the species analyzed, and some properties of their genes.
