# Supplementary material for: Nucleotide composition affects codon usage toward the 3'-end
Source: PLoS One. 2019 Dec 4;14(12):e0225633. doi: 10.1371/journal.pone.0225633 (PMC6892556; doi:10.1371/journal.pone.0225633)
Supplement: S9 Fig — Rows denote species, columns denote positions. Species where none of the codons in a particular efficiency group end by T (missing data) are shown as white stripes. Species within domains are sorted from high (top) to low (bottom) GC-content. (PDF) [file pone.0225633.s009.pdf]

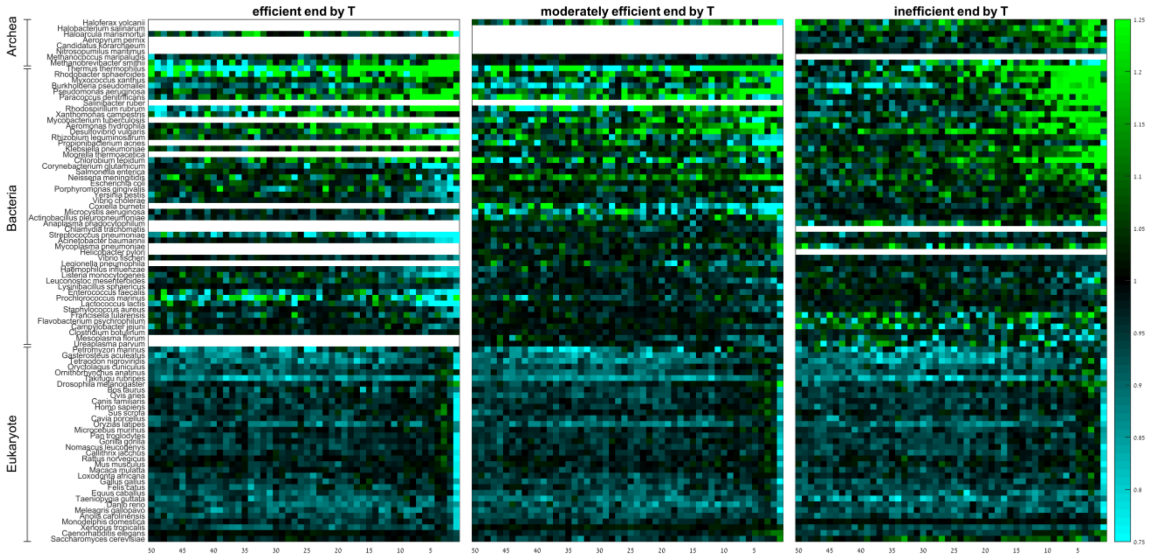

Figure S9. Group RSCA scores (R_{α}^{S}) of efficient, moderately efficient, and inefficient codons ending by T along the last 50 codons of the gene. Rows denote species, columns denote positions. Species where none of the codons in a particular efficiency group end by T (missing data) are shown as white stripes. Species within domains are sorted from high (top) to low (bottom) GC-content.
