# Supplementary material for: Nucleotide composition affects codon usage toward the 3'-end
Source: PLoS One. 2019 Dec 4;14(12):e0225633. doi: 10.1371/journal.pone.0225633 (PMC6892556; doi:10.1371/journal.pone.0225633)
Supplement: S8 Table — (PDF) [file pone.0225633.s022.pdf]

position	1	2	3	4	5	6	7	8	9	10	11	12	13	14	15
Anolis carolinensis	0.87	0.56	0.57	0.09	0.69	0.56	0.87	0.59	0.63	0.56	0.69	0.87	0.36	0.69	0.13
Bos taurus	0.93	0.84	0.93	0.83	0.92	0.84	0.81	0.93	0.81	0.84	0.81	0.81	0.84	0.93	0.83
Caenorhabditis elegans	0.97	0.97	0.97	0.97	0.97	0.97	0.97	0.97	0.97	0.97	0.97	0.97	0.97	0.97	0.97
Callithrix jacchus	0.91	0.81	0.81	0.81	0.85	0.81	0.84	0.91	0.81	0.81	0.81	0.91	0.81	0.93	0.91
Canis familiaris	0.74	0.62	0.62	0.62	0.63	0.62	0.62	0.88	0.62	0.62	0.94	0.36	0.74	0.83	0.62
Cavia porcellus	0.82	0.82	0.89	0.82	0.82	0.89	0.82	0.88	0.83	0.82	0.83	0.82	0.82	0.85	0.82
Danio rerio	0.46	0.19	0.56	0.54	0.15	0.15	0.12	0.41	0.15	0.15	0.15	0.22	0.54	0.46	0.15
Drosophila melanogaster	0.88	0.97	0.40	0.85	0.73	0.40	0.40	0.40	0.40	0.73	0.86	0.40	0.99	0.40	0.86
Equus caballus	0.97	0.87	0.87	0.79	0.79	0.87	0.79	0.91	0.38	0.99	0.87	0.79	0.97	0.79	0.79
Felis catus	0.97	0.87	0.87	0.79	0.79	0.87	0.79	0.91	0.38	0.99	0.87	0.79	0.97	0.79	0.79
Gallus gallus	0.97	0.87	0.87	0.79	0.79	0.87	0.79	0.91	0.38	0.99	0.87	0.79	0.97	0.79	0.79
Gasterosteus aculeatus	0.60	0.60	0.44	0.60	0.65	0.13	0.44	0.68	0.25	0.60	0.44	0.60	0.58	0.66	0.71
Gorilla gorilla	0.77	0.94	0.64	0.74	0.60	0.60	0.64	0.94	0.17	0.94	0.94	0.94	0.94	0.94	0.94
Homo sapiens	0.86	0.86	0.86	0.86	0.86	0.87	0.86	0.87	0.86	0.86	0.86	0.86	0.94	0.86	0.86
Loxodonta africana	0.93	0.93	0.93	0.93	0.93	0.93	0.93	0.93	0.93	0.93	0.93	0.93	0.93	0.93	1.00
Macaca mulatta	0.92	0.85	0.89	0.89	0.76	0.76	0.76	0.76	0.76	0.92	0.92	0.92	0.92	0.92	0.92
Meleagris gallopavo	0.93	0.95	0.95	0.95	0.95	0.93	0.95	0.95	0.95	0.93	0.93	0.93	0.95	0.95	0.95
Microcebus murinus	0.95	0.95	0.95	0.95	0.95	0.95	0.95	0.95	0.95	0.95	0.95	0.95	0.95	0.95	0.95
Monodelphis domestica	0.81	0.81	0.81	0.81	0.81	0.81	0.81	0.89	0.94	0.94	0.98	0.81	0.81	0.81	0.81
Mus musculus	0.96	0.96	0.99	0.96	0.96	0.99	0.99	0.96	0.96	0.96	0.99	0.96	0.96	0.96	0.99
Nomascus leucogenys	0.90	0.76	0.76	0.90	0.76	0.76	0.76	0.93	0.76	0.93	0.93	0.93	0.93	0.93	0.76
Ornithorhynchus anatinus	0.78	0.90	0.78	0.78	0.78	0.78	0.78	0.96	0.97	0.78	0.78	0.97	0.97	0.78	0.96
Oryctolagus cuniculus	0.89	0.95	0.89	0.89	0.89	0.89	0.89	0.89	0.89	0.89	0.89	0.88	0.89	0.89	0.89
Oryzias latipes	0.87	0.47	0.71	0.66	0.66	0.66	0.47	0.66	0.83	0.75	0.66	0.66	0.97	0.71	0.75
Ovis aries	0.94	0.90	1.00	0.90	0.90	0.94	0.90	0.94	0.90	0.90	0.90	0.90	0.90	0.90	0.90
Pan troglodytes	0.74	0.74	0.74	0.74	0.74	0.74	0.74	0.74	0.74	0.74	0.90	0.74	0.74	0.77	0.74
Petromyzon marinus	0.95	0.95	0.95	0.95	0.95	0.97	0.95	0.95	0.95	0.95	0.95	0.95	0.95	0.95	0.95
Rattus norvegicus	0.91	0.91	0.91	0.91	0.91	0.91	0.91	0.91	0.91	0.91	0.91	0.91	0.91	0.91	0.91
Saccharomyces cerevisiae	0.98	0.98	0.98	0.98	0.98	0.98	0.98	0.98	0.98	0.98	0.98	0.98	0.98	0.98	0.98
Sus scrofa	0.99	0.90	0.90	0.90	0.90	0.99	0.90	0.90	0.90	0.99	0.71	0.90	0.90	0.97	0.90
Taeniopygia guttata	0.88	0.59	0.88	0.59	0.78	0.81	0.59	0.78	0.63	0.70	0.81	0.55	0.78	0.78	0.78
Takifugu rubripes	0.85	0.85	0.95	0.85	0.85	0.95	0.07	0.64	0.76	0.85	0.85	0.76	0.85	0.95	0.64
Tetraodon nigroviridis	0.91	0.91	0.91	0.91	0.81	0.91	0.82	0.60	0.52	0.91	0.91	0.91	0.60	0.91	0.81

Xenopus tropicalis	0.42	0.42	0.29	0.33	0.26	0.42	0.43	0.29	0.48	0.30	0.30	0.39	0.53	0.52	0.20
Acinetobacter baumannii	0.77	0.64	0.48	0.39	0.35	0.20	0.64	0.60	0.64	0.64	0.86	0.61	0.84	0.77	0.39
Actinobacillus pleuropneumoniae	0.77	0.66	0.66	0.77	0.53	0.76	0.41	0.74	0.74	0.79	0.95	0.95	0.94	0.66	0.94
Aeromonas hydrophila	0.60	0.60	0.20	0.96	0.60	0.20	0.92	0.93	0.92	0.96	0.96	0.81	0.96	0.96	0.60
Anaplasma phagocytophilum	0.54	0.76	0.76	0.76	0.76	0.76	0.83	0.76	0.76	0.64	0.82	0.76	0.88	0.51	1.00
Burkholderia pseudomallei	0.24	0.24	0.24	0.24	0.24	0.24	0.24	0.26	0.84	0.47	0.45	0.38	0.26	0.28	0.75
Campylobacter jejuni	1.00	0.83	0.96	1.00	0.96	0.96	1.00	0.32	1.00	1.00	1.00	1.00	0.08	1.00	1.00
Chlamydia trachomatis	0.98	0.98	0.71	0.96	0.92	1.00	0.98	0.71	0.98	0.96	0.87	0.96	0.98	1.00	0.71
Chlorobium tepidum	0.31	0.29	0.31	0.39	0.31	0.38	0.31	0.36	0.31	0.30	0.29	0.88	0.38	0.29	0.29
Clostridium botulinum	0.94	0.94	0.94	0.94	0.94	0.94	0.94	0.06	0.94	0.94	0.94	0.94	0.94	0.58	0.94
Corynebacterium glutamicum	0.36	0.37	0.29	0.31	0.15	0.32	0.31	0.32	0.79	0.15	0.15	0.29	0.97	0.60	0.54
Coxiella burnetii	0.62	0.62	0.68	0.56	0.68	0.68	0.68	0.68	0.62	0.62	0.25	1.00	0.99	0.81	0.68
Desulfovibrio vulgaris	0.33	0.33	0.33	0.34	0.34	0.86	0.89	0.79	0.74	0.34	0.79	0.92	0.51	0.34	0.51
Enterococcus faecalis	0.89	0.89	0.89	0.89	0.89	0.89	0.89	0.89	0.89	0.89	0.94	0.89	0.89	0.89	0.94
Escherichia coli	0.13	0.13	0.04	0.04	0.04	0.04	0.04	0.08	0.40	0.67	0.09	0.85	0.52	0.08	0.09
Flavobacterium psychrophilum	0.85	0.85	0.85	0.91	1.00	1.00	1.00	0.85	1.00	0.98	0.85	0.85	1.00	1.00	0.85
Francisella tularensis	0.69	1.00	1.00	0.84	0.54	0.84	1.00	0.84	1.00	0.95	0.84	0.94	1.00	0.54	0.84
Haemophilus influenzae	0.76	0.73	0.73	0.36	0.73	0.73	0.76	0.73	0.76	0.76	0.76	0.73	0.73	0.90	0.90
Helicobacter pylori	0.78	0.79	0.78	0.79	0.78	0.79	0.78	0.95	0.90	0.90	0.78	0.95	0.79	0.80	0.95
Klebsiella pneumoniae	0.39	0.17	0.27	0.11	0.11	0.27	0.27	0.28	0.35	0.35	0.37	0.27	0.53	0.87	0.13
Lactococcus lactis	0.98	0.99	0.97	0.92	0.97	0.98	0.62	0.98	0.99	0.97	0.98	0.98	0.99	0.98	0.99
Legionella pneumophila	0.79	0.99	0.86	0.79	0.79	0.86	0.86	0.99	0.79	0.86	0.86	0.79	0.79	0.86	0.86
Leuconostoc mesenteroides	0.72	0.84	0.84	0.72	0.72	0.72	0.87	0.84	0.22	0.91	0.72	0.84	0.93	0.95	0.72
Listeria monocytogenes	0.66	0.70	0.41	0.70	0.49	0.52	0.35	0.80	0.92	0.92	0.72	0.70	0.54	0.92	0.80
Lysinibacillus sphaericus	0.72	0.85	0.85	0.72	0.41	0.85	0.85	0.87	0.91	0.93	0.93	0.85	0.72	0.93	0.89
Mesoplasma florum	0.93	0.93	0.93	0.93	0.93	0.93	0.93	0.93	0.93	0.94	0.93	0.93	0.93	0.47	0.93
Microcystis aeruginosa	0.55	0.56	0.72	0.65	0.65	0.65	0.22	0.66	0.65	0.22	0.72	0.66	0.80	0.56	0.72
Moorella thermoacetica	0.61	0.27	0.27	0.63	0.27	0.62	0.27	0.82	0.63	0.63	0.27	0.76	0.75	0.27	0.63
Mycobacterium tuberculosis	0.52	0.52	0.46	0.46	0.42	0.45	0.80	0.92	0.52	0.45	0.52	0.75	0.57	0.52	0.52
Mycoplasma pneumoniae	0.95	0.82	0.40	0.93	0.92	0.61	0.99	0.85	0.92	0.71	0.99	0.99	0.40	0.95	0.40
Myxococcus xanthus	0.73	0.73	0.73	0.73	0.93	0.95	0.73	0.73	0.73	0.91	0.73	0.86	0.73	0.93	0.73

Neisseria meningitidis	0.16	0.16	0.34	0.16	0.37	0.20	0.18	0.25	0.18	0.19	0.34	0.25	0.90	0.83	0.37
Paracoccus denitrificans	0.35	0.14	0.27	0.23	0.53	0.27	0.73	0.69	0.36	0.78	0.35	0.69	0.79	0.58	0.27
Porphyromonas gingivalis	0.30	0.30	0.58	0.30	0.43	0.43	0.43	0.71	0.97	0.68	0.97	0.64	0.55	0.43	0.64
Prochlorococcus marinus	0.66	0.62	0.62	0.97	0.97	0.84	0.62	0.66	0.35	0.62	0.97	0.62	0.84	0.62	0.62
Propionibacterium acnes	0.36	0.52	0.65	0.49	0.10	0.61	0.61	0.49	0.36	0.60	0.61	0.96	0.60	0.71	0.96
Pseudomonas aeruginosa	0.47	0.47	0.25	0.47	0.25	0.34	0.53	0.79	0.47	0.79	0.94	0.61	0.99	0.34	0.38
Rhizobium leguminosarum	0.57	0.20	0.11	0.48	0.57	0.33	0.57	0.49	0.77	0.69	0.98	0.98	0.80	0.98	0.57
Rhodobacter sphaeroides	0.54	0.29	0.34	0.29	0.92	0.64	0.64	0.67	0.64	0.90	0.97	0.64	0.42	0.90	0.29
Rhodospirillum rubrum	0.56	0.56	0.56	0.79	0.40	0.56	0.84	0.56	0.56	0.84	0.56	0.98	0.98	0.84	0.97
Salinibacter ruber	0.37	0.95	0.84	0.79	0.74	0.84	0.84	0.84	0.84	0.97	0.74	0.92	0.74	0.79	0.84
Salmonella enterica	0.18	0.13	0.14	0.10	0.10	0.10	0.13	0.53	0.13	0.60	0.91	0.37	0.91	0.10	0.30
Staphylococcus aureus	0.42	0.93	0.89	0.65	0.78	0.65	0.42	0.78	0.98	0.62	0.78	0.42	0.65	0.98	0.65
Streptococcus pneumoniae	0.91	0.91	0.91	0.91	0.91	0.91	0.91	0.91	0.91	0.91	0.91	0.91	0.91	0.91	0.91
Thermus thermophilus	0.60	0.88	0.56	0.46	0.56	0.80	0.89	0.46	0.76	0.85	0.46	0.95	0.46	0.90	0.80
Ureaplasma parvum	0.68	0.99	0.68	0.68	0.66	0.68	0.78	0.87	0.68	0.68	0.88	0.88	0.68	0.68	0.78
Vibrio cholerae	0.36	0.19	0.60	0.14	0.14	0.14	0.60	0.36	0.60	0.75	0.38	0.60	0.44	0.82	0.74
Vibrio fischeri	0.70	0.73	0.44	0.73	0.36	0.44	0.23	0.99	0.96	0.70	0.77	0.44	0.77	0.44	0.52
Xanthomonas campestris	0.40	0.89	0.32	0.25	0.40	0.69	0.89	0.78	0.93	0.78	0.69	0.69	0.69	0.72	0.69
Yersinia pestis	0.40	0.89	0.32	0.25	0.40	0.69	0.89	0.78	0.93	0.78	0.69	0.69	0.69	0.72	0.69
Aeropyrum pernix	0.30	0.30	0.91	0.36	0.91	0.30	0.36	0.97	0.91	0.84	0.91	0.73	0.91	0.91	0.70
Candidatus korarchaeum	0.53	0.52	0.87	0.87	0.87	0.87	0.87	0.81	0.94	0.87	0.87	0.87	0.52	0.69	0.52
Haloarcula marismortui	0.40	0.64	0.64	0.57	0.21	0.57	0.64	0.79	0.94	0.64	0.64	0.21	0.81	0.93	0.95
Halobacterium salinarum	0.57	0.89	0.93	0.90	0.95	0.89	1.00	0.93	0.99	1.00	0.51	0.93	1.00	0.93	1.00
Haloferax volcanii	0.64	0.69	0.93	0.93	0.69	0.69	0.93	0.93	0.98	0.93	0.69	0.69	0.93	0.94	0.94
Methanobrevibacter smithii	0.99	0.99	0.99	0.81	0.99	0.99	0.99	0.99	0.99	0.99	0.99	0.99	0.99	0.70	0.99
Methanococcus maripaludis	0.99	0.99	0.99	0.99	0.91	0.99	0.99	0.99	0.99	0.91	0.99	0.91	0.99	0.91	0.99
Nitrosopumilus maritimus	1.00	0.95	1.00	0.95	0.95	1.00	0.95	0.95	1.00	1.00	0.95	0.95	0.95	0.96	1.00

Table S8. For each position (distance from the stop codon, columns) and for every species, we computed the Kruskal-Wallis (FDR corrected) p-value, measuring the difference in the distribution of RSCA values between replaceable, non-replaceable, and non-fragile that end by A/T.
