# Supplementary material for: Nucleotide composition affects codon usage toward the 3'-end
Source: PLoS One. 2019 Dec 4;14(12):e0225633. doi: 10.1371/journal.pone.0225633 (PMC6892556; doi:10.1371/journal.pone.0225633)
Supplement: S10 Table — (PDF) [file pone.0225633.s024.pdf]

Species	Median expected MFE	Median observed MFE	p-value
Anolis carolinensis	-9.8	-7.6	2.150e-38
Bos taurus	-9.4	-7.3	2.755e-48
Caenorhabditis elegans	-8.4	-6.1	1.622e-72
Callithrix jacchus	-9.4	-7.4	6.827e-40
Canis familiaris	-10.5	-8.2	7.198e-41
Cavia porcellus	-9.7	-7.7	5.008e-32
Danio rerio	-9.1	-6.9	1.324e-42
Drosophila melanogaster	-8.8	-7.3	2.928e-45
Equus caballus	-9.6	-7.7	7.464e-37
Felis catus	-9.9	-8.0	2.241e-38
Gallus gallus	-9.7	-7.7	5.803e-44
Gasterosteus aculeatus	-10.2	-8.8	4.017e-11
Gorilla gorilla	-10.1	-7.7	3.359e-48
Homo sapiens	-9.8	-8.4	1.500e-18
Loxodonta africana	-9.4	-7.8	1.895e-24
Macaca mulatta	-9.5	-7.6	3.644e-41
Meleagris gallopavo	-10.0	-8.8	2.748e-12
Microcebus murinus	-9.6	-7.7	4.077e-43

Monodelphis domestica	-9.4	-7.1	3.484e-46
Mus musculus	-9.8	-8.0	1.783e-21
Nomascus leucogenys	-9.9	-7.7	3.664e-39
Ornithorhynchus anatinus	-9.3	-7.5	3.728e-24
Oryctolagus cuniculus	-11.0	-9.5	5.332e-21
Oryzias latipes	-9.8	-8.6	1.177e-24
Ovis aries	-9.9	-7.6	2.909e-47
Pan troglodytes	-10.0	-8.0	1.045e-32
Petromyzon marinus	-10.1	-8.9	3.406e-13
Rattus norvegicus	-9.7	-7.6	8.945e-52
Saccharomyces cerevisiae	-7.9	-5.3	1.212e-83
Sus scrofa	-9.4	-7.3	1.952e-33
Taeniopygia guttata	-9.9	-8.2	2.798e-19
Takifugu rubripes	-10.1	-8.5	2.567e-22
Tetraodon nigroviridis	-10.1	-8.3	6.676e-44
Xenopus tropicalis	-8.9	-6.5	1.533e-56
Acinetobacter baumannii	-8.5	-5.9	2.989e-81
Actinobacillus	-8.8	-6.1	2.674e-94

pleuropneumoniae			
Aeromonas hydrophila	-12.2	-11.3	3.487e-21
Aeropyrum pernix	-11.6	-9.6	4.811e-66
Anaplasma phagocytophilum	-8.6	-6.6	6.184e-64
Burkholderia pseudomallei	-14.8	-14.0	4.810e-10
Campylobacter jejuni	-7.2	-4.1	2.281e-106
Candidatus korarchaeum	-10.7	-8.3	1.040e-81
Chlamydia trachomatis	-8.8	-6.1	1.640e-68
Chlorobium tepidum	-10.4	-8.8	1.211e-54
Clostridium botulinum	-6.9	-3.6	2.173e-103
Corynebacterium glutamicum	-10.4	-8.8	1.449e-50
Coxiella burnetii	-8.7	-6.1	1.676e-81
Desulfovibrio vulgaris	-12.8	-11.2	1.465e-37
Enterococcus faecalis	-8.2	-4.9	1.545e-108
Escherichia coli	-10.3	-8.4	6.433e-69
Flavobacterium psychrophilum	-6.7	-3.5	8.408e-111

Francisella tularensis	-7.2	-4.5	1.006e-99
Haemophilus influenzae	-8.6	-5.7	1.258e-91
Haloarcula marismortui	-11.6	-9.8	1.051e-48
Halobacterium salinarum	-12.1	-10.6	7.539e-31
Haloferax volcanii	-12.9	-11.8	1.195e-19
Helicobacter pylori	-7.9	-5.1	2.902e-91
Klebsiella pneumoniae	-11.1	-9.1	2.711e-67
Lactococcus lactis	-7.8	-4.8	1.545e-108
Legionella pneumophila	-8.2	-5.4	4.305e-101
Leuconostoc mesenteroides	-7.7	-5.1	1.044e-81
Listeria monocytogenes	-7.8	-4.6	9.034e-106
Lysinibacillus sphaericus	-8.1	-5.0	1.117e-111
Mesoplasma florum	-6.9	-3.5	4.396e-80
Methanobrevibacter smithii	-7.4	-4.3	1.888e-106
Methanococcus maripaludis	-7.4	-4.0	4.694e-115

Microcystis aeruginosa	-8.6	-5.9	1.444e-84
Moorella thermoacetica	-11.1	-9.2	5.245e-78
Mycobacterium tuberculosis	-13.5	-12.5	8.686e-31
Mycoplasma pneumoniae	-7.8	-5.2	8.711e-67
Myxococcus xanthus	-14.5	-13.9	6.959e-05
Neisseria meningitidis	-10.3	-8.0	4.961e-77
Nitrosopumilus maritimus	-7.7	-4.5	8.049e-107
Paracoccus denitrificans	-14.0	-12.9	3.271e-31
Porphyromonas gingivalis	-9.2	-6.9	7.527e-87
Prochlorococcus marinus	-8.2	-5.2	1.847e-101
Propionibacterium acnes	-11.9	-10.6	1.852e-25
Pseudomonas aeruginosa	-13.9	-12.9	2.532e-20
Rhizobium leguminosarum	-12.5	-10.9	1.791e-40
Rhodobacter	-14.4	-13.6	4.976e-14

sphaeroides			
Rhodospirillum rubrum	-13.5	-12.6	3.375e-25
Salinibacter ruber	-13.1	-12.4	2.054e-22
Salmonella enterica	-10.4	-8.6	2.345e-60
Staphylococcus aureus	-7.3	-4.1	1.450e-107
Streptococcus pneumoniae	-8.4	-5.5	8.344e-86
Thermus thermophilus	-15.0	-14.3	4.446e-29
Ureaplasma parvum	-6.4	-3.3	1.415e-65
Vibrio cholerae	-9.5	-7.4	1.062e-62
Vibrio fischeri	-8.4	-5.8	7.876e-85
Xanthomonas campestris	-13.7	-13.0	2.095e-18
Yersinia pestis	-9.9	-7.7	3.353e-62

Table S10. Median minimum free energy (MFE) at the 3'-end (observed MFE), median MFE expected by the global (not spatial) codon bias (expected MFE), and p-value for testing whether the median difference between pairs of expected and observed values equals zero (FDR-corrected two-sided sign test).
